# *Naegleria*’s mitotic spindles are built from unique tubulins and highlight core spindle features

**DOI:** 10.1016/j.cub.2022.01.034

**Published:** 2022-02-08

**Authors:** Katrina B. Velle, Andrew S. Kennard, Monika Trupinić, Arian Ivec, Andrew J.M. Swafford, Emily Nolton, Luke M. Rice, Iva M. Tolić, Lillian K. Fritz-Laylin, Patricia Wadsworth

**Affiliations:** 1 Department of Biology, University of Massachusetts, 611 N. Pleasant Street, Amherst, MA 01003, USA; 2 Division of Molecular Biology, Ruđer Bošković Institute, Bijenićka cesta 54, 10000 Zagreb, Croatia; 3 Department of Physics, Faculty of Science, University of Zagreb, Bijenićka cesta 32, 10000 Zagreb, Croatia; 4 Departments of Biophysics and Biochemistry, UT Southwestern Medical Center, Dallas, TX 75390, USA; 5 These authors contributed equally; 6 Twitter: @fritzlaylin; 7 Lead contact

## Abstract

*Naegleria gruberi* is a unicellular eukaryote whose evolutionary distance from animals and fungi has made it useful for developing hypotheses about the last common eukaryotic ancestor. *Naegleria* amoebae lack a cytoplasmic microtubule cytoskeleton and assemble microtubules only during mitosis and thus represent a unique system for studying the evolution and functional specificity of mitotic tubulins and the spindles they assemble. Previous studies show that *Naegleria* amoebae express a divergent α-tubulin during mitosis, and we now show that *Naegleria* amoebae express a second mitotic α- and two mitotic β-tubulins. The mitotic tubulins are evolutionarily divergent relative to typical α- and β-tubulins and contain residues that suggest distinct microtubule properties. These distinct residues are conserved in mitotic tubulin homologs of the “brain-eating amoeba” *Naegleria fowleri*, making them potential drug targets. Using quantitative light microscopy, we find that *Naegleria*’s mitotic spindle is a distinctive barrel-like structure built from a ring of microtubule bundles. Similar to those of other species, *Naegleria*’s spindle is twisted, and its length increases during mitosis, suggesting that these aspects of mitosis are ancestral features. Because bundle numbers change during metaphase, we hypothesize that the initial bundles represent kinetochore fibers and secondary bundles function as bridging fibers.

## In brief

*Naegleria* amoebae are profoundly different from other eukaryotes as they lack interphase microtubules. During cell division, Velle et al. show that *Naegleria* express α- and β-tubulins that are highly divergent at key structural positions. These tubulins form spindles with an unusual architecture: a ring of twisted microtubule bundles.

## Graphical abstract

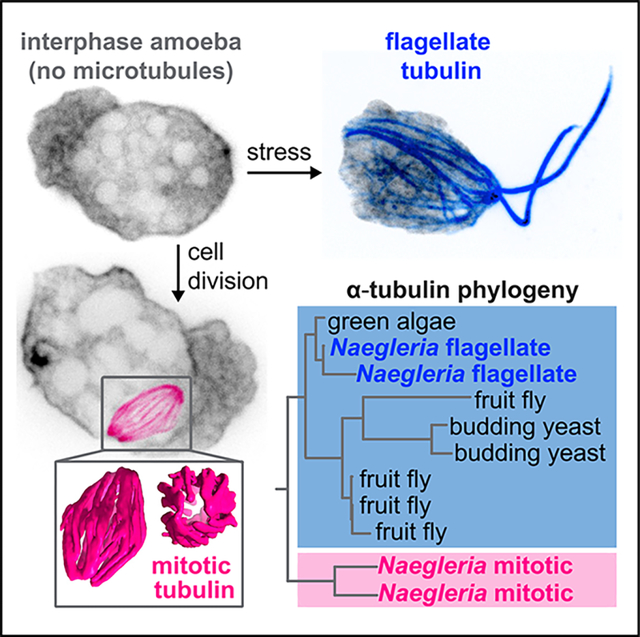

## INTRODUCTION

Cells from across the eukaryotic tree use microtubules for a variety of functions during both interphase and mitosis. Interphase microtubules contribute to cell shape, polarity, and intracellular trafficking. During cell division, a microtubule-based spindle mediates chromosome segregation.^1,2^ Interphase and mitotic microtubule functions are emergent properties of microtubule-associated proteins as well as the subunit composition and post-translational modifications of tubulin. Eukaryotic cells typically express multi-functional tubulins used for both interphase and mitotic functions.^3^ For example, human embryonic kidney cells express high levels of one α-tubulin and two β-tubulins (80% identical),^4^ while budding yeast express one β-tubulin and two α-tubulins (88% identical), and each uses these tubulins in both interphase and mitosis.^5^ As an extreme example, the unicellular algae *Chlamydomonas* has one α- and one β-tubulin gene that are used for all microtubule functions.^6^ Other eukaryotes, however, express unique tubulin isotypes for specific functions, including meiotic spindle assembly in *Drosophila* oocytes,^7^ axoneme formation in diverse systems,^8^ and touch receptor neurons in worms.^9^ These specialized tubulins support the “multi-tubulin hypothesis” that posits that different tubulins can specify distinct cellular functions.^10,11^

**Figure 1.**
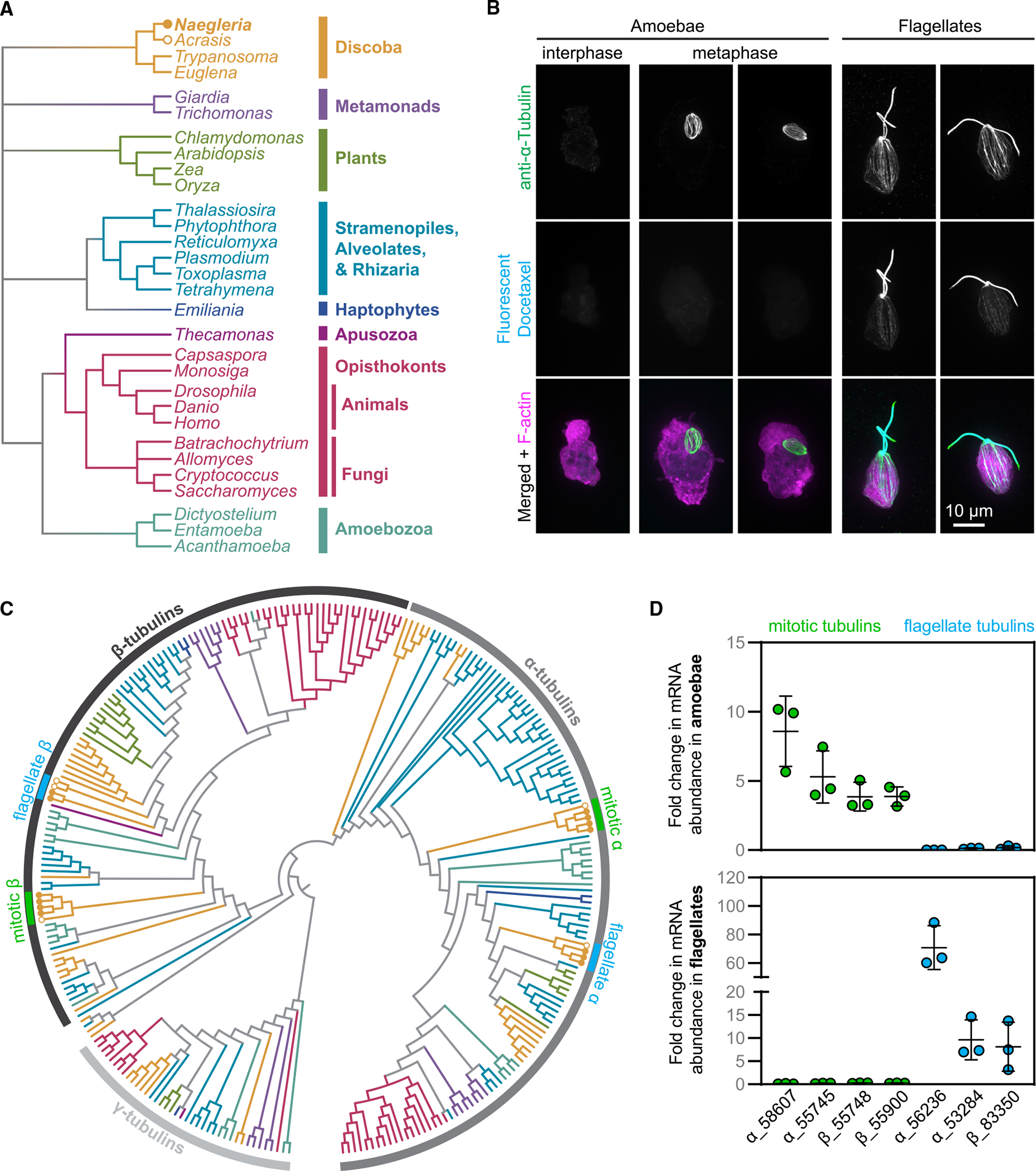
*Naegleria* has flagellate and mitotic microtubule arrays composed of distinct tubulins (A) The evolutionary relationships between *Naegleria* and other eukaryotes are shown using a cladogram (branch lengths are meaningless) modified from Velle and Fritz-Laylin.^15^ (B) Amoebae from a growing population (left) or flagellates from a differentiated population (right) were fixed and stained with antibodies (anti-α-tubulin clone DM1A, green) and tubulin Tracker (fluorescent docetaxel, cyan) to detect microtubules and Alexa Fluor 488 conjugated Phalloidin to label F-actin (magenta). Maximum intensity projections of cells are shown. (C) The evolutionary relationship of γ-, α-, and β-tubulins from the species in (A) are shown using a cladogram (using the color scheme from A; see Data S2 for the full tree). The tree is rooted on gamma tubulins, and shows mitotic (green) and flagellate (blue) tubulins from *Naegleria* (closed circles) and *Acrasis* (open circles). (D) The fold changes in tubulin mRNA in amoebae compared with flagellates (top) or flagellates compared with amoebae (bottom) were calculated from data reported in Fritz-Laylin and Cande (from 3 experimental replicates encompassing 6 technical replicates).^16^ Each point represents one experimental replicate, and lines denote the average ± standard deviation (SD). Tubulins are labeled with JGI identification numbers. See also Figure S1, Data S1, S2, S3, S4, S5, and S6, and Tables S2 and S3.

*Naegleria gruberi* is a single-celled eukaryote that diverged from the “yeast-to-human” lineage over a billion years ago (Figure 1A) with the unusual ability to differentiate from a crawling amoeba to a swimming flagellate (Figure 1B).^12^ This stress response involves the assembly of an entire microtubule cytoskeleton—centrioles, flagella, and a cortical microtubule array—including transcription and translation of flagellate-specific α- and β-tubulins along with associated microtubule-binding proteins.^13^ The flagellate form is transient, and cells return to crawling amoebae within 2–300 min,^14^ after which the flagellate microtubules are disassembled and tubulin is degraded. The *Naegleria* flagellate microtubules, and the α- and β-tubulins that comprise them, are specific for these non-mitotic microtubule functions, an idea that stimulated the development of the multi-tubulin hypothesis.^11^

Unlike other eukaryotes, interphase *Naegleria* amoebae lack tubulin transcripts^17,18^ and have *no* observable microtubules as visualized by immunofluorescence (Figure 1B),^19,20^ or electron microscopy.^21^ *Naegleria* amoebae, however, do assemble microtubules within the nucleus for closed mitosis.^19–22^ Previous studies have shown that *Naegleria* expresses a divergent α-tubulin specifically during mitosis^18^ that is incorporated into the mitotic spindle.^18–20^ *Naegleria*, therefore, represents a unique test of the multi-tubulin hypothesis.

The most well-studied spindles are those of animal cells, which contain functionally distinct populations of microtubules, including (1) kinetochore fiber microtubules that bind to kinetochores to connect each chromosome to a single spindle pole;^23^ (2) non-kinetochore microtubules that extend from the poles and overlap at the midzone, linking the two halves of the spindle;^24–27^ and (3) astral microtubules that extend from spindle poles toward the cell cortex. During anaphase, kinetochore microtubules shorten (anaphase A),^28^ while midzone microtubules elongate to drive chromosome segregation (anaphase B).^29^ A subset of midzone microtubules, called bridging fibers, closely approach kinetochore fibers in each half spindle.^30^ Bridging fibers contribute to the balance of tension and compressive forces in the spindle,^30^ chromosome alignment, and chromosome motion in anaphase.^31–33^ Spindle microtubules are organized by mitotic motor proteins that promote microtubule dynamic turnover, spindle pole organization, chromosome congression during prometaphase, and poleward motion in anaphase.^34^ The influence of motor proteins in spindle structure is highlighted by the twist they introduce in spindles of human cell lines.^35^

Outside of animals, there exists a wide diversity of spindle architecture and molecular mechanisms driving chromosome segregation.^36^ While some organisms break down the nuclear envelope to facilitate microtubule-chromosome interaction (open mitosis), others nucleate microtubules in the cytoplasm that pass through holes in the nuclear envelope to interact with chromosomes (semi-open mitosis), or, like *Naegleria*, assemble microtubules within an intact nuclear envelope (closed mitosis).^19,20,37^ Spindle-microtubule-organizing centers also vary widely, from centriole-containing centrosomes that nucleate spindle microtubules in the cytoplasm of animal cells, to spindle pole bodies that nucleate mitotic microtubules from the surface of the nuclear envelope in yeast, to diffuse microtubule-organizing centers in land plants, as well as a wide variety of microtubule-organizing centers and spindle architectures found in protist lineages.^38–41^ Despite this wide diversity of spindle organization, eukaryotic chromosome segregation generally requires three activities: (1) a regularly structured, microtubule-based spindle apparatus. No eukaryotic species has yet to be reported that does not use microtubules to segregate its chromosomes, and each species assembles a characteristic spindle structure prior to mitosis. (2) Chromosome interaction with microtubules. This usually occurs via attachment of kinetochores to the ends of microtubules as in cultured mammalian cells,^23^ or lateral interactions as in *C*. *elegans* meiosis.^42^ (3) Microtubule dynamics. Mitotic microtubules are nucleated and grow to form the spindle and are subsequently disassembled after chromosome segregation.

In line with its unusual α-tubulin, the architecture of the *Naegleria* spindle is also unconventional; *Naegleria*’s spindle is barrel-shaped and lacks obvious microtubule-organizing centers and homologs of many proteins found in conventional kinetochores.^21,22,43–46^ Here, we test whether, in the absence of the evolutionary constraints imposed by interphase microtubule functions, *Naegleria*’s mitotic microtubule system has diverged from canonical systems. We show that, in addition to the previously reported mitotic α-tubulin, *Naegleria* expresses a second mitotic α-tubulin along with two mitotic β-tubulins. In contrast to the *Naegleria* tubulins expressed during the flagellate stage that closely resemble tubulins from heavily studied species, the protein sequences of the *Naegleria* mitotic tubulins have diverged significantly, consistent with the original multi-tubulin hypothesis.^11^ We demonstrate that mitotic tubulins are used to build an unusual spindle composed of a ring of regularly spaced microtubule bundles that twists end-to-end. As mitosis proceeds, additional microtubule bundles form in the equatorial region of the spindle and—as in other eukaryotes—the spindle elongates to facilitate chromosome segregation. The organization and dynamics of the *Naegleria* spindle highlight both core aspects of mitosis as well as variable features of cell division.

## RESULTS

### *Naegleria* expresses divergent α- and β-tubulins during mitosis

To determine the number and diversity of tubulins available to *Naegleria* amoebae and flagellates, we searched for α- and β-tubulins in the *Naegleria gruberi* genome.^47^ As previously reported, we identified 13 α- and 9 β-tubulin genes, some of which appeared highly divergent, while others are closely related to those of other eukaryotes.^47^ To further explore the diversity of *Naegleria* tubulins, we reconstructed a phylogenetic tree of α- and β-tubulins using γ-tubulins as an outgroup. Briefly, we collected and aligned 1,191 tubulin sequences from 200 different species (Table S3; Data S1), reconstructed a maximum likelihood tree (Data S2 and S3) and pruned the resulting tree to visualize the sequences of interest (Figure 1C; Data S4). The tree recovers α-tubulins and β-tubulins as two, monophyletic clades with *Naegleria* mitotic and flagellar tubulins forming evolutionarily distinct clades within each tubulin family (Figure 1C).

The *Naegleria* α- and β-tubulin sub-clades most closely related to animal and fungal tubulins include those that are expressed during differentiation to the flagellate form.^16,17,48^ These tubulins represent the majority of axonemal and cytoplasmic tubulin protein in flagellates^49–51^ and are not expressed in amoebae.^16,17,48^ Flagellate α-tubulins are 79%–85% identical to human α-tubulin A1B (ENSP00000336799) and flagellate β-tubulins are 74%–75% identical to human β-tubulin B1 (ENSP00000217133) (Figure S1E).

The second *Naegleria* tubulin sub-clades are more divergent. The second clade of α-tubulins contains two sequences each from *Naegleria gruberi* and *Naegleria fowleri* and one from each of the related species *Acrasis kona* and *Stachyamoeba lipophora*. The two *N. gruberi* α-tubulins are only 57%–58% identical to human α-tubulin A1B (Figure S1E). Similarly, the second clade of *Naegleria* β-tubulins also includes *N. fowleri* and *A. kona* sequences, with *N. gruberi* sequences that are 57%–58% identical to human β-tubulin B1.

Because the ortholog of the previously reported mitotic α-tubulin (from the NB-1 strain) was among the divergent α-tubulins (from strain NEG-M),^18,47^ we predicted that the divergent *Naegleria* α- and β-tubulins are expressed during mitosis. Consistent with this prediction, we compared expression data of amoebae (a population that includes dividing cells) and flagellates and found that, while the conserved tubulins are expressed in flagellates, the divergent tubulins are expressed in amoebae (Figure 1D). We confirmed this by comparing expression levels of the putative-mitotic tubulins in mitotically synchronized cells with control populations and found at least 2-fold enrichment of the divergent tubulin transcripts (Figures S1A and S1B). Our transcriptional data are in line with previous measurements by immunoblotting of mitosis-specific expression of one divergent α-tubulin isoform,^18^ although protein levels of the other isoforms—and hence the composition of tubulin heterodimers—have not been investigated. Together these data indicate that *Naegleria gruberi* amoebae express divergent α- and β-tubulins during cell division that are conserved in fellow heterolobosean species *N. fowleri* and *A. kona*.

### *Naegleria* mitotic tubulins have diverged in ways that suggest distinct biochemical properties

**Figure 2.**
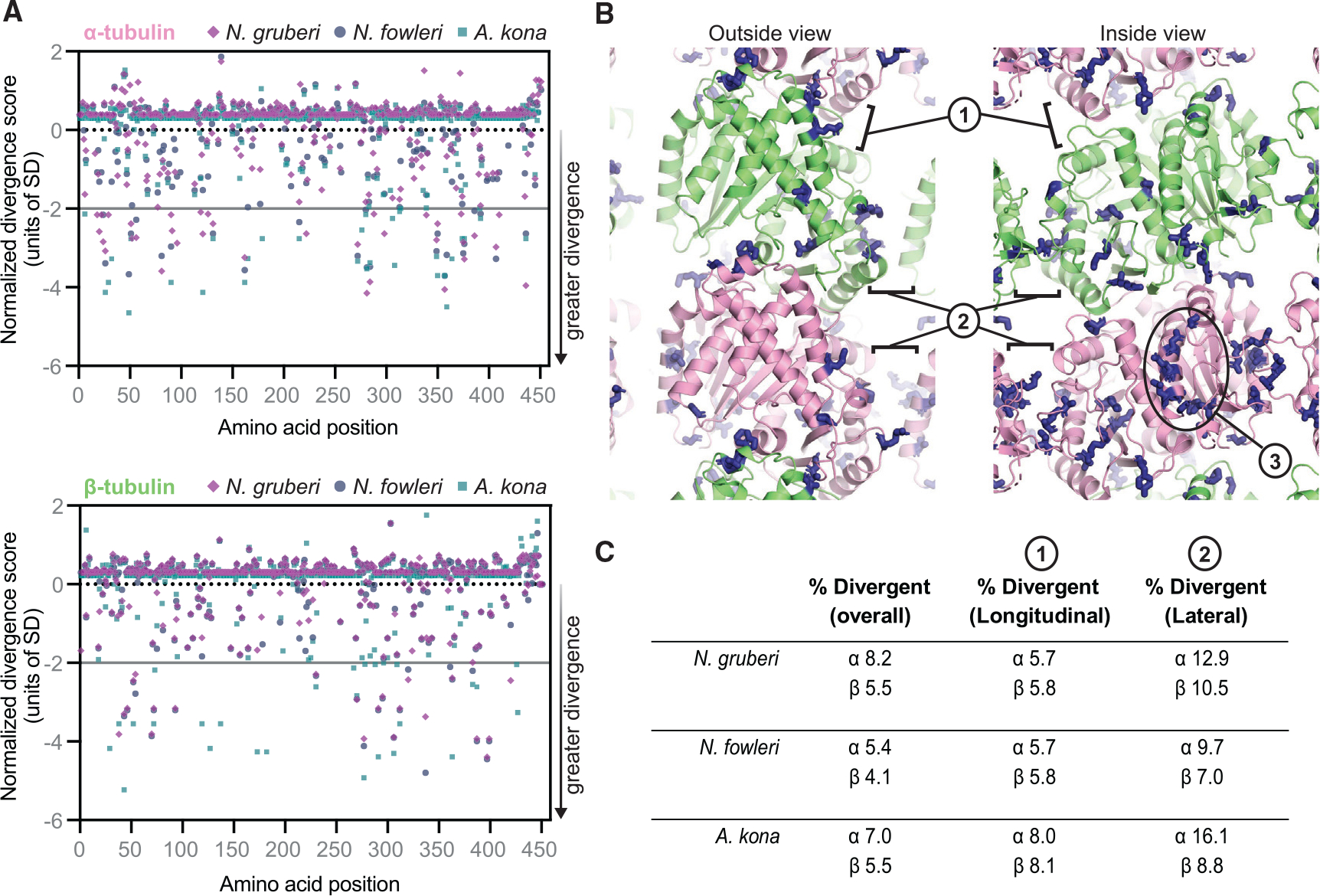
Comparative analysis of evolutionary divergence for mitotic and flagellate tubulins (A) Plots of the normalized divergence score (STAR Methods) as a function of amino acid position for α-tubulin (top) and β-tubulin (bottom). Lower scores indicate positions where mitotic tubulins show increased divergence relative to flagellate tubulins. The analysis was performed on three species: *N. gruberi* (lavender diamonds), *N. fowleri* (navy circles), and *A. kona* (teal squares). The horizontal gray line indicates the two standard deviation cutoff we used to identify especially divergent sites. (B) Structural context of the sites with increased divergence in the mitotic tubulins. Side chain positions for the *N. gruberi* amino acids identified in (A) are represented as sticks (blue) on a model of αβ-tubulin in the microtubule lattice (α-tubulin, pink; β-tubulin, lime). “Outside” and “inside” views of the lattice are shown, and longitudinal (labeled 1) and lateral (labeled 2) microtubule lattice contacts are indicated, as is the luminal (internal) surface of α-tubulin (labeled 3). (C) Table summarizing the proportion of positions with elevated divergence near microtubule lattice interfaces. For all three species, there are more divergent positions in α-tubulin compared with β-tubulin, and the divergence seems to be particularly enriched at the lateral interfaces. See also Figures S2 and S3, Data S5 and S6, and Table S2.

Inspection of *Naegleria* mitotic and flagellate tubulin sequences suggested that the mitotic tubulins may have altered microtubule dynamics and/or binding sites for microtubule-associated proteins. To assess this possibility, we quantified the divergence of mitotic and flagellate α- and β-tubulins as a function of amino acid position. Briefly, after building master multiple sequence alignments for α- and β-tubulins from *N. gruberi*, *N. fowleri*, and *A. kona* along with reference sequences from more commonly studied organisms, we made separate “mitotic” and “flagellate” subalignments for each species by only retaining the mitotic or flagellate tubulin from that species (in addition to the reference sequences). We used these subalignments to measure the relative conservation at each position and summarized the results with a positional “divergence score” (Figure 2A) in which lower scores indicate positions where mitotic tubulins show increased divergence relative to flagellate tubulins. Mitotic α-tubulins have more positions with elevated divergence compared with β-tubulin in all three species (compare Figure 2A, top and bottom), although the absolute number of divergent positions differs by organism (35 positions in α-tubulin versus 23 in β-tubulin for *N. gruberi*; 24 versus 22 for *N. fowleri*; 32 versus 27 for *A. kona*).

Although the positions of elevated variability are distributed throughout the tubulin fold for both α- and β-tubulin, they appear to be enriched near microtubule polymerization interfaces and interior microtubule surfaces (Figures 2B, S2A, and S2B). To quantify this impression, we tested for enrichment at longitudinal or lateral polymerization interfaces by determining whether the fraction of divergent positions near a given interface was greater than the fraction of divergent positions across the entire sequence. This analysis reveals that divergent positions are more enriched at lateral lattice contacts (2- to 3-fold increase depending on the species) than at longitudinal lattice contacts (1.1- to 1.9-fold, depending on the species; Figure 2C). Some of the substitutions are striking and likely to have substantial effects on tubulin-tubulin interactions. For example, a tryptophan residue (β-tubulin W397, human B1 sequence numbering) that participates in longitudinal contacts and that is invariant in the reference and flagellate tubulin sequences is mutated to a much smaller and less hydrophobic residue (serine or threonine) in the mitotic tubulin sequences from *N. gruberi* and *N. fowleri*. Likewise, a glutamate residue (α-tubulin E90, human A1A sequence numbering) that likely forms a salt bridge (with α-tubulin K280, human A1A sequence numbering) at the lateral interface and that is strongly conserved in the reference and flagellate tubulin sequences is mutated to smaller, uncharged residues (alanine, asparagine, serine, or glycine) in all mitotic sequences examined. The sidechain character can also change significantly at other positions (see Data S5 and S6 for alignments). This enrichment of divergence at lattice interfaces of *N. gruberi, N. fowleri,* and *A. kona* reinforces the idea that microtubules formed from mitotic tubulins will have altered polymerization dynamics and/or distinct structural features.

Because fluorescent docetaxel—a reagent derived from the microtubule-stabilizing drug taxol—appears to bind *Naegleria* flagellate tubulin but not mitotic tubulin (Figure 1B), we next examined if taxol-binding residues in β-tubulin^52,53^ were conserved in either of these *Naegleria* sequences. Important taxol-binding amino acids are conserved in flagellate but not in mitotic β-tubulin sequences (Figure S2C). Furthermore, we observed little-to-no growth defects for *Naegleria* grown in the presence of high concentrations of a variety of conventional microtubule inhibitors, including nocodazole, colchicine, vinblastine, and oryzalin (Figures S1C and S1D), suggesting that these drugs may not bind mitotic microtubules.

Finally, we observed key sequence differences in disordered regions of the *Naegleria* tubulins. For example, the major site of α-tubulin acetylation, K40, is conserved in the flagellate tubulins but has diverged in the mitotic tubulins (Figure S2D). We also characterized the length and predicted net charges of the C-terminal tubulin tails (Figure S2E); while the tubulin tails of both mitotic and flagellate α-tubulins have lengths and net charges similar to commonly studied tubulins, the mitotic β-tubulin tails are slightly less charged than their flagellate counterparts (Figure S2E). Moreover, the C-terminal EY sequence in α-tubulin that is recognized by regulatory factors that contain a CAP-Gly domain is notably absent from both flagellate and mitotic α-tubulin sequences. This is surprising given our previous identification of two *Naegleria* genes with CAP-Gly motifs (protein IDs 81169 and 51258 from of Fritz-Laylin et al.^47^), both of which are induced during the amoeba-to-flagellate transition.^16^ In fact, the only heterolobosean tubulins that end in a C-terminal tyrosine are the β flagellar tubulins (Figure S2E). Together with the lack of C-terminal tyrosines in *Naegleria* EB1 homologs (protein IDs 44546 and 65633 from Fritz-Laylin et al.^47^), these data hint that the CAP-Gly proteins could bind directly to the flagellar β-tubulins, a hypothesis that awaits verification.

To further investigate the divergent properties of *Naegleria* mitotic tubulins, we stained cells with antibodies against several post-translational tubulin modifications. We observed robust staining of flagellate microtubules but no staining of mitotic spindles with the anti-acetylated tubulin antibody 6–11B-1 (Figure S3A), consistent with the presence of the K40 residue in *Naegleria* flagellate but not mitotic tubulins. Corroborating the lack of the C-terminal tyrosine in *Naegleria* α-tubulins, we did not observe staining of flagellates or mitotic amoebae with an antibody specific for C-terminally tyrosinated α-tubulin (YL1/2; Figure S3A). Finally, we did not observe any structures in *Naegleria* amoebae or flagellates that were stained by antibodies against poly-glutamylation modifications that mark centrosomes in mammalian cells (Figure S3A). Together, these observations reinforce the notion that microtubules assembled from mitotic αβ-tubulins of *N. gruberi*, *N. fowleri*, and *A. kona* are likely to have different polymerization dynamics and/or binding partners compared with microtubules assembled from flagellate αβ-tubulins.

### The *Naegleria* spindle is a barrel of microtubule bundles that elongates as mitosis proceeds

**Figure 3.**
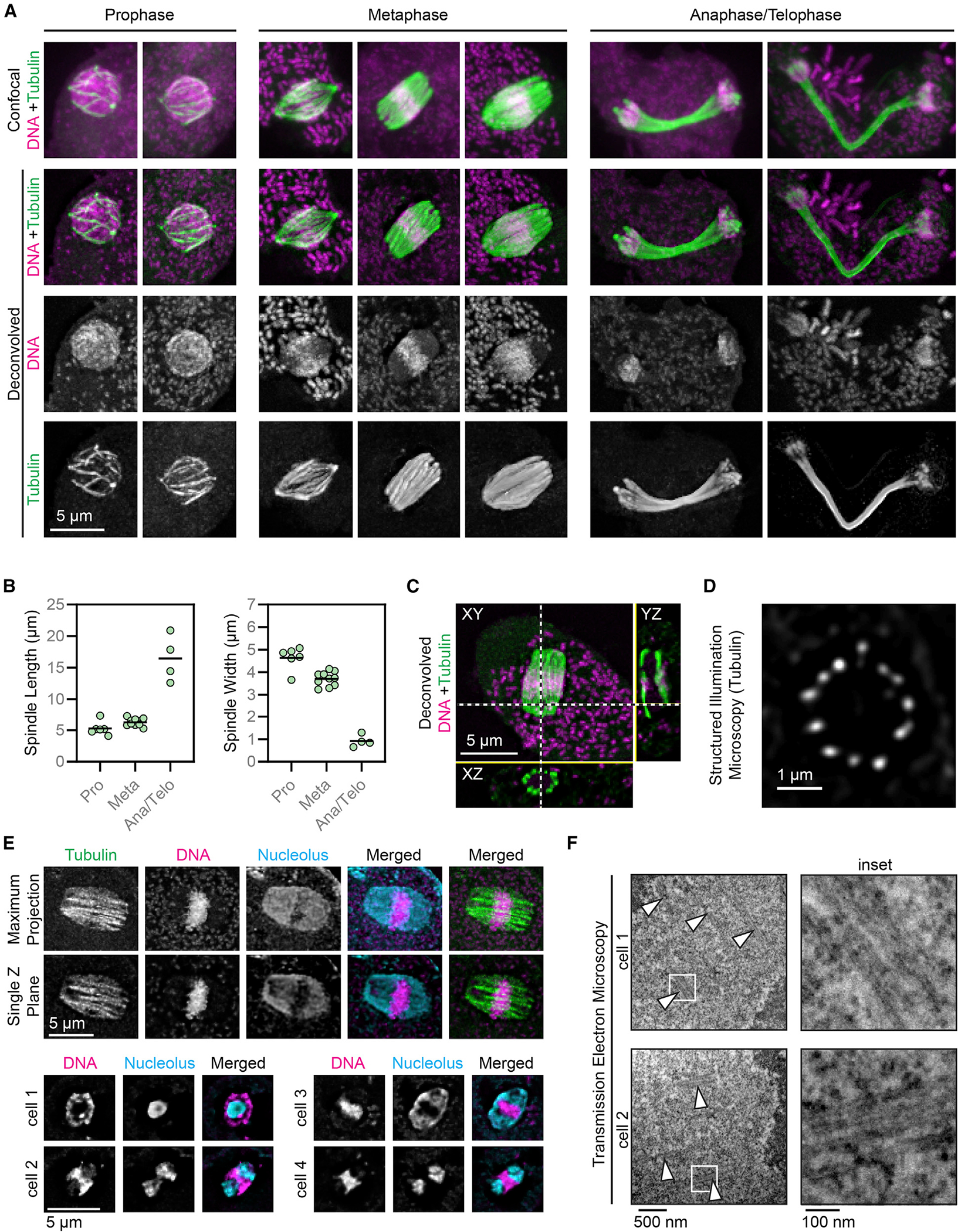
*Naegleria*’s spindle is a barrel shape composed of bundles of microtubules that elongate as mitosis proceeds (A) Asynchronously growing *Naegleria* amoebae were fixed and stained with anti-α-tubulin clone DM1A (green) to detect microtubules and DAPI to label DNA (magenta). Mitotic spindles were imaged using confocal microscopy (top row), and images were deconvolved using Autoquant software (bottom rows). Cells were classified as prophase, metaphase, or anaphase/telophase. (B) Quantification of maximum spindle length (left) and the spindle width at half the length (right). Each point represents one mitotic spindle, and lines indicate the averages (from 3 experimental replicates encompassing the following numbers of cells: prophase, n = 6; metaphase, n = 10; anaphase/telophase, n = 4). Spindles were imaged and deconvolved as in (A). (C) Orthogonal views of a metaphase spindle (imaged and deconvolved as in A) lying in the plane of the coverslip; XZ and YZ views generated in Fiji. (D) Structured illumination microscopy of a spindle lying perpendicular to the coverslip. (E) Confocal microscopy and deconvolution of nucleoli in mitotic *Naegleria*. Cells were fixed and stained to detect tubulin (YOL 1/34 antibody, green, top panels only), DNA (DAPI, magenta), and nucleolar protein (DE6 antibody, cyan). One maximum intensity projection is shown (top cell), while remaining images are single z planes. (F) Transmission electron microscopy of microtubule bundles in *Naegleria*; arrowheads indicate microtubule bundles and boxed regions (left) are shown as enlarged insets (right). See also Figures S3–S5 and Table S2.

To explore whether the sequence divergence of *Naegleria*’s mitotic tubulins translates into a divergent spindle organization, we stained the microtubules of fixed amoebae with anti-tubulin antibodies and DNA with DAPI (which also prominently stains *Naegleria*’s mitochondrial DNA; Figures S3B and S3C).^18,20,21,54^ Consistent with work showing that *Naegleria* mitotic microtubules assemble within the intact nucleus, but not from a single point on the nuclear envelope,^18,20–22,54^ we find that the *Naegleria* spindle is composed of microtubule bundles and lacks obvious microtubule-organizing centers (Figure 3). The microtubule bundles appear to form around a ball of DNA; we refer to this stage as prophase. This cage-like array of microtubule bundles reorganizes into a barrel-shaped spindle with DNA aligned in a broad band at the midplane; we refer to this stage as metaphase. Although in some cases the spindle has a tapered morphology (Figure 3A, left metaphase cell), most spindles are characterized by broad, flat poles (Figure 3A, middle and right metaphase cells; see Figure S4C for analysis).

We also observed spindles in which the DNA is segregated to the ends of the elongated spindle, which we classified as anaphase/telophase. Relatively few spindles were detected showing early stages of chromosome segregation, suggesting that this stage is short lived. In contrast, cells with elongated spindles and segregated DNA were relatively common, suggesting that late anaphase spindles persist for some time. Moreover, spindle length increases while width decreases as mitosis progresses from prophase to anaphase/telophase (Figure 3B). The localization of chromosomes near the ends of anaphase spindles, along with the increased length of anaphase versus metaphase spindles, indicates the presence of both anaphase-A-like chromosome segregation and anaphase-B-like spindle elongation, although the timing and duration of these processes cannot be determined from fixed cells.

Because mitotic cells were relatively rare in asynchronous populations, we also examined mitotically synchronized cells^55^ and found no qualitative or quantitative differences between them (Figures S4A–S4E). This supports previous reports that synchronization does not alter spindle morphology in *Naegleria* amoebae.^55^ We therefore used cells from both synchronized and asynchronized populations for the following analyzes.

To determine the organization of microtubule bundles in the *Naegleria* spindle, we visualized axial and transverse slices of spindles oriented both parallel and perpendicular to the coverslip. These analyses confirmed that metaphase spindle microtubules are organized in a ring, similar to the staves of a barrel (Figures 3C and 3D). Previous studies have suggested that this barrel is assembled around the nucleolus, which remains intact during mitosis (*Naegleria*’s ribosomal RNA genes are encoded on a plasmid that does not condense during prophase^20,54^). To confirm the retention of the nucleolus during mitosis, we costained cells with anti-nucleolar and/or anti-tubulin antibodies, as well as DAPI to visualize DNA (Figure 3E). Consistent with previous work, we find that the nucleolus remains visible throughout mitosis, at times encompassing much of the spindle volume.^20^ The nucleolus divides before chromosome segregation, resulting in one nucleolus at each end of the spindle with the chromosomes nestled between them (Figure 3E).

Comparing the dimensions and intensity of the microtubule arrays in flagellates with those in mitotic cells suggests that the spindle is composed of bundles rather than individual microtubules (Figure 1B), consistent with previous findings.^21,56^ Supporting this idea, we observed a single anaphase cell in which a microtubule bundle appears to have splayed apart, revealing at least five fluorescent elements which may represent individual microtubules (Figure S4F). To estimate the number of microtubules per bundle, we fixed *Naegleria* amoebae for transmission electron microscopy (TEM). Longitudinal sections through mitotic cells reveal that bundles are composed of three to six closely associated microtubules (Figures 3F and S5), in accordance with previous estimates in *N. fowleri*.^56^ Consistent with previous TEM data,^21^ none of these sections contained electron-dense material between the microtubules and the nuclear envelope (Figure S5). In summary, our data show that the *Naegleria* spindle is composed of a ring of microtubule bundles that elongates during chromosome segregation and lacks obvious microtubule-organizing structures.

### *Naegleria* spindles have two sets of microtubule bundles

**Figure 4.**
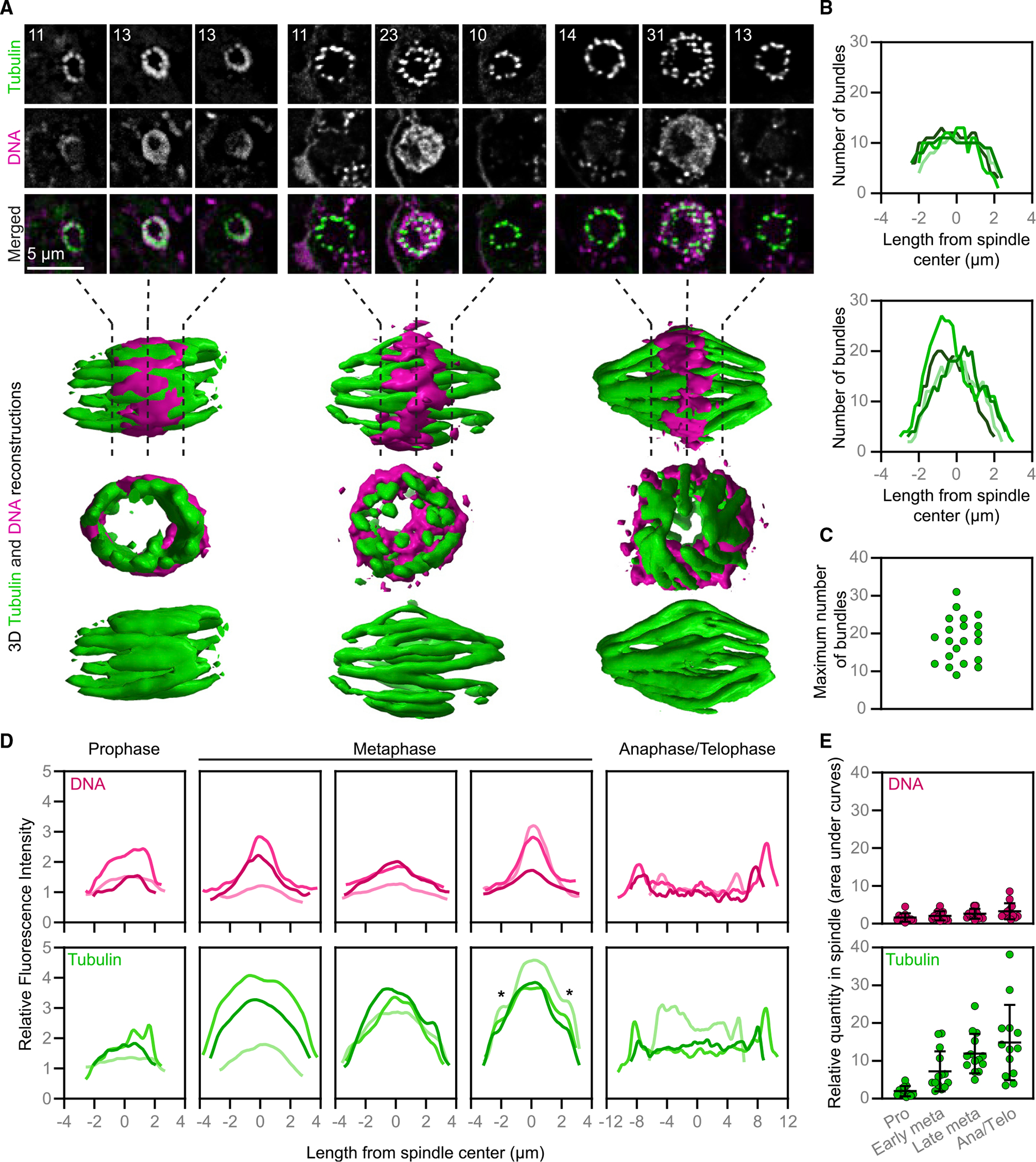
The number of microtubule bundles changes as mitosis proceeds (A) Cells were fixed and stained with antibodies (anti-α-tubulin clone DM1A, green) to detect microtubules and DAPI to label DNA (magenta). Cells with spindles perpendicular to the coverslip were imaged using confocal microscopy and deconvolved using Autoquant software (top panels), and 3D reconstructions were rendered using ChimeraX software (bottom panels, not to scale). Individual z planes are shown for slices approximately 25%, 50%, and 75% through the spindle for three representative cells. Numbers (upper left) indicate the number of distinct microtubule bundles in that position of the spindle. (B) The number of microtubule bundles throughout the spindle length in metaphase spindles, imaged as in (A). Some spindles (top) had a fairly consistent number of microtubule bundles throughout the spindle, while other spindles (bottom) had a peak in the number of bundles toward the midpoint. Each line represents one spindle, pooled from three experimental replicates. (C) The maximum number of microtubule bundles from confocal images of metaphase cells (calculated from 4 experimental replicates encompassing 21 cells). Each point represents one cell. (D) Line scans show the relative DNA and tubulin fluorescence intensity from sum intensity projections of spindles lying in the plane of the coverslip, imaged as in (A). Metaphase spindles were grouped based on the shapes of tubulin curves (no shoulders, left; unclear shoulders, center; two clear shoulders denoted by asterisks, right); three individual examples are shown in each panel (also see Figures S4G and S4H). Each line represents one spindle; a total of 15 representative spindles were selected from 25 analyzed images from two experimental replicates. (E) Quantification of DNA (top) or tubulin (bottom) from line scans obtained as in (D). Metaphase was categorized as early or late based on the presence (late) or absence (early) of shoulders (stages where no clear classification could be assigned were excluded). Each point represents the area under the curve for one spindle line scan, and lines indicate the mean ± SD. Values were calculated from 52 spindles, pooled from 4 experimental replicates. See also Figures S4 and S6, Videos S1 and S2, and Table S2.

Although most spindles were oriented parallel to the coverslip surface, some were perpendicular, providing improved resolution (Figure 4A) and revealing variation in the number of microtubule bundles (Figures 4A–4C). Some spindles have a single ring of ~12 evenly spaced bundles with 0.79 μm center-to-center spacing (range: 0.42–1.90; SD: 0.28; n = 31 measurements from 3 spindles). These “primary bundles” extend the entire length of the spindle (Figures 4A, left and 4B, top). Other spindles, however, have additional bundles adjacent to the main ring (Figure 4A, middle and right and 4B, bottom). Importantly, these secondary bundles were restricted to the spindle midplane and did not extend to the spindle poles.

If the secondary bundles were formed from new microtubule polymerization, we would expect the mid-region of metaphase spindles to have a greater amount of tubulin than the poles. We therefore quantified tubulin and DNA fluorescence intensity along horizontally oriented spindles at each stage of mitosis (Figures 4D, S4G, and S4H). The total amount of tubulin within the spindle increases as mitosis proceeds, consistent with microtubule assembly (Figure 4E). Metaphase spindles show variable tubulin distributions (Figure 4D), with a subset having a clear peak of intensity toward the spindle midzone with “shoulders” on either side (Figure 4D, rightmost metaphase). This pattern is consistent with the larger number of bundles that we quantified at the centers of vertically oriented spindles (Figure 4B), and with secondary bundle formation involving additional microtubule assembly. Although this subset of metaphase spindles had clear “shoulders” in their tubulin distributions, other distributions were less clear-cut (Figure 4D, center metaphase panel). The variability in the tubulin distribution along metaphase spindles raises the possibility that secondary bundles form asynchronously within a spindle, consistent with cross sections of vertically oriented spindles that show few secondary bundles (Figure 4A, middle cell). We also found that the maximum number of bundles in vertically oriented spindles varies from ~10 to 30, with many cells showing intermediate values (Figure 4C). This continuous distribution is consistent with asynchronous secondary bundle assembly rather than the two distinct populations we would expect for a synchronous event. To distinguish the spindles at each end of this distribution, we use the term “early metaphase” for spindles with a single set of primary microtubule bundles and “late metaphase” for spindles with primary and obvious secondary microtubule bundles (Figure 4E).

To determine the fate of the secondary bundles that form during metaphase, we examined tubulin distribution in anaphase and telophase. Although the tubulin intensity was relatively uniform across the spindle midzone, we observed distinct peaks at each end of the spindle, indicating a higher density of microtubules (Figure 4D, anaphase/telophase), consistent with both primary and secondary bundles remaining associated with chromosomes throughout mitosis. Together, these data suggest that secondary bundles assemble asynchronously during metaphase and persist through late mitosis.

### The *Naegleria* spindle twists from pole-to-pole in a right-handed fashion

**Figure 5.**
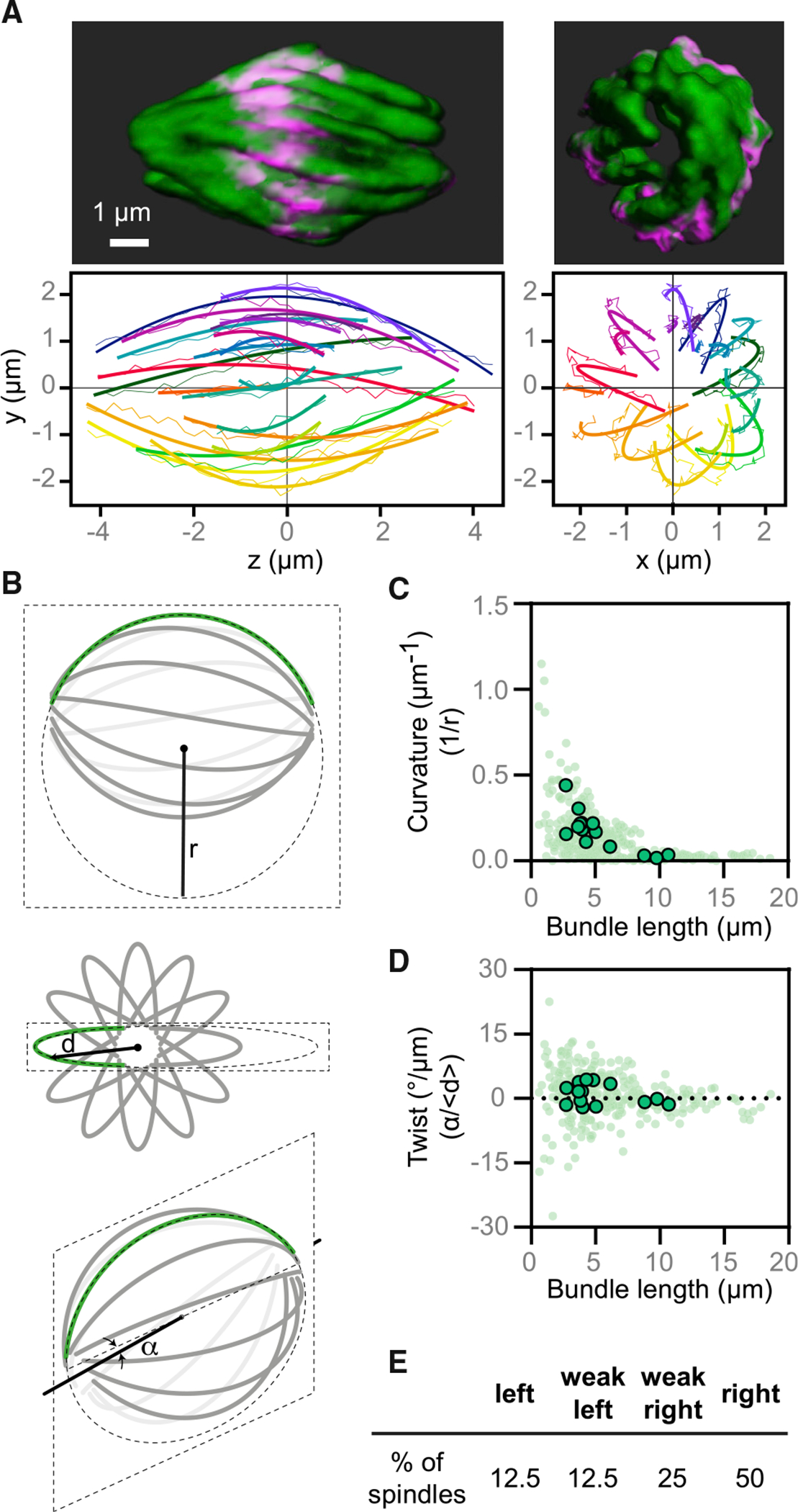
*Naegleria* mitotic spindles are twisted (A) A 3D reconstructed spindle (the same spindle shown in Figure 4A, right) is shown from side and end-on view viewpoints. Microtubules are shown in green, and DNA is in magenta. Microtubule bundles were quantified from the side view (left graph) and end-on view (right graph). Each bundle is represented by a different color, thin lines mark the manually traced points along the bundle, and thick lines show circular arcs of the fitted circles. (B) A simplified scheme of a spindle is shown from the side (top), end-on (middle), and from an arbitrary angle (bottom). A microtubule bundle (green line) is fitted by a circle (dashed ellipse) of radius (r). The angle (α) between the central spindle axis (solid line) and the plane in which the fitted circle lies (dashed parallelogram) is denoted. The distance (d) of the bundle from the central spindle axis is denoted. (C) The curvature of microtubule bundles is shown as a function of bundle length (measured along its pole-to-pole axis). Each small dot represents a single bundle within a spindle, while each larger dot represents the average for a spindle. (D) The twist of microtubule bundles is shown as a function of bundle length. Each small dot represents a single bundle within a spindle, while each larger dot represents the average for a spindle. The data in (C) and (D) are from 4 experimental replicates, encompassing 14 cells and 301 bundles. (E) The percentage of spindles with right, weak right, left, or weak left handedness are shown (see Figure S6 for a breakdown of this analysis). Data were analyzed for 40 cells from 4 experimental replicates. See also Figure S6, Video S1, and Tables S1 and S2.

The 3D reconstructions of vertically oriented spindles revealed that the microtubule bundles curved and appeared to twist from one end of the spindle to the other (Figures 4A and 5A; Videos S1 and S2). Such twist has so far been documented only in HeLa, U2OS, and hTERT-RPE1 cells, where it is generated through the activity of the spindle kinesins Eg5/kinesin-5 and Kif18A/kinesin-8 and regulated by other microtubule-binding proteins.^35,57,58^ To quantify the degree of twist in the *Naegleria* spindle, we traced individual metaphase bundles (Figure 5A) and measured their curvature and twist by fitting a plane to the points representing the bundle and a circle that lies in this plane to the same points. We then estimated bundle curvature as the inverse of the radius of the fit circle, and the twist as the angle between the plane and the z axis divided by the mean distance of these points from the z axis (Figure 5B).

The resulting data show that microtubule bundles in the *Naegleria* spindle are curved (0.146 ± 0.009/μm, Figure 5C) and twisted (0.873° ± 0.316°/μm; positive values denote right-handed and negative values left-handed twist Figure 5D), with shorter bundles having more curve and twist than longer bundles (Figures 5C and 5D). This result was corroborated by visual assessment of the handedness of the spindle twist (if the bundles rotate counterclockwise when moving along the spindle axis toward the observer, the twist is right-handed). We found a mixture of left- and right-handed twist, with the majority of spindles showing a strong right-handed twist (Figure 5E). Analyzing early metaphase (defined for this analysis as cells with <20 bundles) separate from late metaphase (cells with >20 bundles) suggests that bundles increase in length and decrease in curvature during metaphase (Figures S6A and S6D). Right-handed twist was dominant for vertically and horizontally oriented spindles and for cells in early and late metaphase (Figures S6B and S6G), suggesting that the handedness of spindle chirality does not depend on mitotic stage or spindle orientation during imaging.

The microtubule bundles of the *Naegleria* spindle are less curved than those of HeLa cells, as the radius of curvature is larger for *Naegleria*, 6.9 ± 0.4 μm, than for the outermost bundles in HeLa cells, 5.1 ± 0.3 μm.^59^ Moreover, the radius of curvature normalized to the spindle half-length, which is equal to 1 for bundles shaped as a semicircle, is 1.26 ± 0.05 for *Naegleria* and 0.90 ± 0.05 for HeLa cells,^59^ also indicating a smaller curvature of *Naegleria* spindles. In line with the smaller curvature, the absolute value of the average spindle twist in *Naegleria* is smaller than in HeLa cells, 0.9° ± 0.3°/μm in *Naegleria* versus 2°/μm in HeLa.^35^ The twist of *Naegleria* spindles is more eye-catching than in HeLa cells, however, due to the smaller number of microtubule bundles, which are well defined and have a uniform shape, in contrast to the less ordered distribution and shapes of bundles in HeLa cells. Together, these data indicate that the microtubule bundles that comprise the *Naegleria* spindle are physically linked and under rotational forces.

## DISCUSSION

*Naegleria* amoebae represent a remarkable system for studying cytoskeletal regulation because they do not have interphase microtubules. *Naegleria* is not the only species without interphase microtubules; the cytoplasm of interphase Entamoeba histolytica amoebae also has no observable microtubules.^60^ In contrast to *Entamoeba*, however, *Naegleria* can differentiate into a secondary cell type, the flagellate. Here, we show that *Naegleria* express unique tubulins in mitotic amoebae that are distinct from the tubulins expressed in flagellate cells. While flagellate tubulins—used to assemble both flagellar and cytoplasmic microtubules in flagellates^11,16,49,61,62^—are highly similar to tubulins of other eukaryotes, the mitotic tubulins have diverged at key residues likely to alter microtubule structure and/or dynamics. Because the sequence similarity between *Naegleria* and *Acrasis* flagellate tubulin isoforms is much higher than between the mitotic tubulins of these species (Figure S1E), we infer that the cytoplasmic functions of tubulins may require more stringent sequence conservation than mitotic functions.

Previous fluorescent microscopy showed that *Naegleria* spindle assembly begins with the formation of microtubule bundles that rearrange to form a barrel-shaped spindle that elongates as mitosis progresses.^20^ Electron microscopy showed that microtubule bundles in the spindles of both *N. gruberi* and *N. fowleri* are composed of several microtubules.^21,56^ The exclusive use of microtubule bundles differentiates the *Naegleria* spindle from those of other species that typically contain both single and bundled microtubules.^25^ Our higher resolution imaging extends these observations, demonstrating that the spindle is composed of ~12 primary bundles arranged in a ring, with additional bundles incorporated as mitosis progresses, and an obvious pole-to-pole twist.

**Figure 6.**
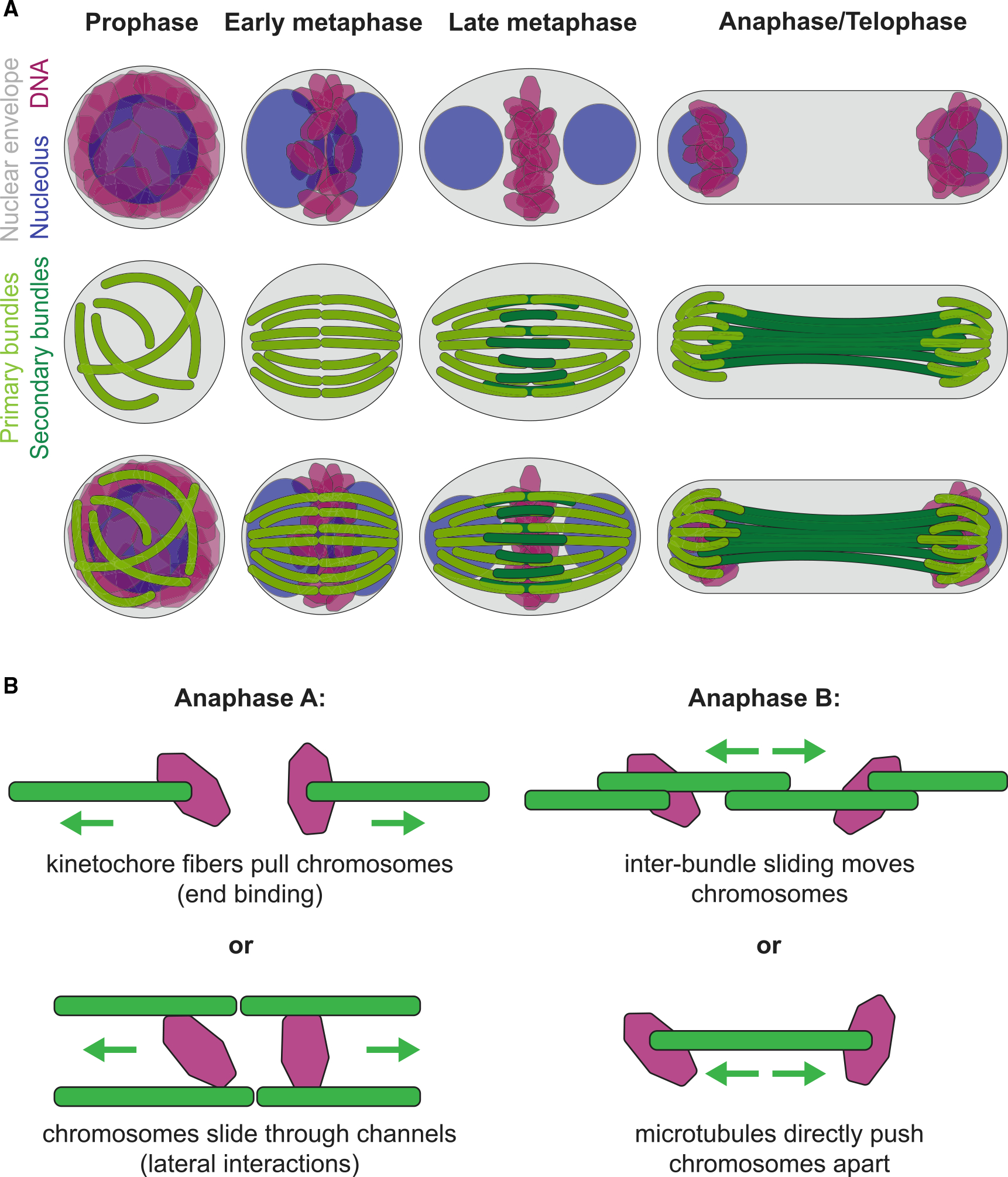
Model for mitosis in *Naegleria* (A) During prophase in *Naegleria*, bundles of microtubules form around a hollow sphere of DNA (magenta), which surrounds the single, round nucleolus (blue). In early metaphase, the DNA condenses, the nucleolus begins to divide, and the microtubule bundles (light green) organize into a hollow, twisted barrel shape. In late metaphase, the DNA is further condensed, and the nucleolus resolves into two distinct spheres. A secondary set of microtubules forms in the equatorial region (dark green) adjacent to the primary bundles. During anaphase/telophase, the DNA is segregated to the two ends of the spindle and the spindle elongates. See text for details. (B) Insets show different possible mechanisms for chromosome separation in anaphase A (left) and anaphase B (right).

Based on our data, we infer that *Naegleria* mitosis proceeds through the following four stages (Figure 6A): (1) mitosis begins with the assembly of disorganized microtubule bundles surrounding a ball of DNA; (2) “primary” microtubule bundles eventually form a ring with DNA at the midplane; (3) during metaphase, “secondary” microtubule bundles form near the chromosomes at the spindle midplane; and (4) chromosome-to-pole motion occurs as the spindle elongates. Based on this series of events, there are multiple possible mechanisms underlying chromosome motion in anaphase A and anaphase B of *Naegleria* mitosis, several of which we discuss here (Figure 6B).

In many organisms, metaphase chromosomes first move toward the spindle poles in anaphase A using microtubules attached end-on to chromosomes. As these “kinetochore fibers” shorten, they pull sister chromosomes toward opposite ends of the spindle. In *Naegleria*, we observe short microtubule bundles at both ends of anaphase spindles (Figures 3A and S4A), consistent with anaphase-A-like microtubule disassembly (Figure 6B). Moreover, previous estimates of ~12 chromosomes in *Naegleria*^47^ match the ~12 primary bundles we observe in early metaphase spindles (Figure 4), which is tantalizing, albeit indirect, evidence for the hypothesis that primary bundles act as kinetochore fibers in *Naegleria*. Furthermore, we observe “kinks” in the center of some spindles suggesting that each primary bundle may be composed of a pair of kinetochore fibers (e.g., Figure 4A, right spindle). Explicit tests of this model will require appropriate antibodies to localize these elements within the spindle; although *Naegleria* kinetochores and centromere sequences have yet to be identified,^21^ the *Naegleria* genome encodes homologs of a subset of canonical kinetochore proteins, including the centromeric histone CENP-A.^43,46^ Alternatively, anaphase A in *Naegleria* mitosis might proceed similarly to *C. elegans* meiosis, wherein chromosomes interact laterally with microtubules during anaphase A to move through channels formed by the microtubule bundles (Figure 6B).^63,64^ Until more is known about the geometry of the interaction of *Naegleria* chromosomes with microtubules, it will be difficult to distinguish between these two models.

Spindle elongation during anaphase B can also drive chromosome segregation and is typically mediated by polymerization and sliding of antiparallel midzone microtubules. Recent work in several human cell lines has shown that a subset of spindle midzone microtubules, called bridging fibers, overlap in the spindle midplane and closely approach kinetochore fibers in each half spindle. These bridging fibers link sister kinetochores and contribute to both chromosome alignment during prometaphase and spindle elongation in anaphase.^31–33^ As in many other species, *Naegleria*’s anaphase/telophase spindles are longer than metaphase spindles (Figures 3, S4B, and S4D), consistent with anaphase B being driven by spindle elongation. Furthermore, the secondary bundles of *Naegleria* spindles assemble in the spindle midplane in metaphase, elongate in anaphase, and are positioned near primary bundles, consistent with a bundle-bundle sliding mechanism. Color-coded 3D reconstructions of *Naegleria* spindles indicate that primary and secondary bundles come into close proximity, raising the possibility of bundle-bundle interactions (Figures S6I and S6J). We therefore hypothesize that secondary bundles may function as bridging fibers in the *Naegleria* spindle contributing to spindle elongation.

Although our data are consistent with microtubule elongation and sliding driving chromosome segregation in anaphase B, other mechanisms are also possible. In anaphase B of *C. elegans* meiosis, for example, polymerizing midzone microtubules push chromosomes further apart but do not extend beyond the chromosomes (Figure 6B).^42,64^ This contrasts with our observation of additional tubulin near the poles of anaphase *Naegleria* spindles (Figure 4D). For this reason, we favor a model in which secondary bundles function similarly to bridging fibers and contribute to spindle elongation by interactions with primary microtubule bundles rather than direct interactions with chromosomes (Figure 6B).^65^

Similar to results from human cell lines,^35,57,58^ the microtubule bundles in *Naegleria* spindles twist. This observation implies that *Naegleria*’s mitotic microtubule bundles are physically connected, a hypothesis that may explain their regular spacing within the spindle. The function of spindle chirality in human cells may be a passive mechanical response to spindle forces that decreases the risk of spindle breakage under high load.^58,66^ In contrast to the left-handed chirality observed in human cell lines,^35,57,58^ the majority of *Naegleria* spindles are right-handed. When hTERT-RPE1 cells are depleted of components of the key spindle regulator augmin, the spindle twist reverses and becomes right-handed,^58^ indicating that the chirality of twist is modulated by microtubule-associated proteins. Intriguingly, *Naegleria* lacks homologs of the entire augmin complex,^47^ in line with the reversed chirality of *Naegleria* spindles relative to that of augmin-expressing hTERT-RPE1 cells.

Because spindle chirality in these human cell lines requires kinesin-5 (Eg5) and kinesin-8 (Kif18A) motor activity, we hypothesize that *Naegleria* spindle twist also relies on mitotic motor-generated torque.^35,58^ In support of this idea, we mined previous transcriptional analyzes of *Naegleria* differentiation^16^ and found several kinesins whose expression was up to 8-fold enriched in asynchronously dividing amoebae compared with non-dividing flagellates, including homologs of spindle-associated kinesin-5 and kinesin-14 (Table S1).

*Naegleria*’s evolutionary position makes it well suited for identifying features of mitotic spindles that may be deeply conserved, including their bi-polarity, elongation, and twist. *Naegleria*’s position also highlights features that may be lineage specific due to their absence in this distant species. For example, features of animal cell spindles that are missing from *Naegleria* include obvious microtubule-organizing centers and astral microtubules that contribute to spindle position and to cytokinesis.

Moreover, the unique properties of *Naegleria* mitotic tubulins may have practical value. Although *Naegleria gruberi* is innocuous, the related *Naegleria fowleri* is the infamous “brain-eating amoeba” that causes a devastating and usually lethal infection.^67^ Because the divergent residues we have identified in the *Naegleria* mitotic tubulins are conserved in both *Naegleria* species but not in human tubulins (Figures 2 and S2C–S2E), these residues represent specific, potential targets for therapeutics to disrupt *Naegleria* cell division and growth.

## STAR★METHODS

### RESOURCE AVAILABILITY

#### Lead Contact

Further information and requests for resources and reagent should be directed to and will be fulfilled by the lead contact, Lillian Fritz-Laylin (lfritzlaylin@umass.edu)

#### Materials Availability

This study did not generate new unique reagents.

#### Data and Code Availability

All data are available in the figures, tables, and data files associated with this manuscript. This paper does not report original code. Any additional information required to reanalyze the data reported in this paper is available from the lead contact upon request.

### EXPERIMENTAL MODEL AND SUBJECT DETAILS

*Naegleria* amoebae (strain NEG) and their food source *Aerobacter aerogenes* (a gift from the laboratory of Chandler Fulton, Brandeis University) were routinely cultured following previously established protocols.^12^ Briefly, *A. aerogenes* were regularly streaked from a frozen glycerol stock, and single colonies were grown stationary at room temperature in penassay broth (Difco antibiotic medium 3). Liquid cultures were used to grow lawns of *A. aerogenes* overnight on NM plates (2 g/L Gibco Bacto peptone, 2 g/L glucose, 1.5 g/L K2HPO4, 1 g/L KH2PO4, 20 g/L agar). Lawns were inoculated with a loopful of NEG amoebae or cysts to create an edge plate (from a previous edge or cyst plate). Plates were sealed with parafilm, inverted, and incubated for 1–3 days at 28 °C. For starvation-induced differentiation (Figures 1B and S3A), cells were shocked with ice cold 2 mM Tris, and transferred to a shaking flask at 28 °C for 1 h.

Axenic *Naegleria gruberi* amoebae (strain NEG-M) were grown in M7 medium (0.362 g/L KH2PO4, 0.5 g/L Na2HPO4, 5.4 g/L glucose, 5 g/L yeast extract (Difco), 45 mg/L L-methionine, 10% fetal bovine serum) at 28 °C without shaking in 25 cm^2^ plug-seal tissue culture flasks (CellTreat Cat#229330).

### METHOD DETAILS

#### Phylogenetic tree estimation

To establish a more inclusive comparison of *Naegleria* α-, and β-tubulins to those of other eukaryotes, 1,191 tubulins from 200 different species were analyzed (Table S3), adding sequences from *Naegleria gruberi*,^47^ *Naegleria fowleri*,^77^ and *Acrasis kona* (S. Baldauf, personal communication) to those identified as α, β, and γ tubulins using the PhyloToL pipeline.^68^ Prior to alignment, sequences from the same species that were 100% identical were removed, leaving only one copy before re-merging the datasets. Sequences were aligned using the PASTA iterative alignment algorithm with the MUSCLE algorithm as the aligner and merger.^69^ IQ-Tree v1.16.2 was used for model selection, which indicated LG4M+R10 as the best model for reconstruction.^70,78^ Due to the size of the tree, LG4M was used to balance the accuracy of tree solving and the constraints of modern processing power. A maximum likelihood tree was reconstructed using IQ-Tree with 10,000 ultrafast bootstraps.^79^ 1,000 bootstraps of the approximate likelihood ratio test^80^ as well as the aBayes test^81^ were then used to further test node support. The ITOL web server was used for tree visualization.^71^

#### Characterization of mitotic tubulin sequences

To quantify the divergence of mitotic and flagellate α- and β-tubulins from *N. gruberi*, *N. fowleri*, and *A. kona* as a function of amino acid position, we compared them to a common reference alignment consisting of α- or β-tubulin sequences from commonly studied model organisms (*Homo sapiens, Sus scrofa, Bos taurus, Drosophila melanogaster, Mus musculus, Saccharomyces cerevisiae, Schizosaccharomyces pombe, and Chlamydomonas reinhardtii*). Multiple sequence alignments were first prepared for α- and β-tubulin using ClustalOmega.^72^ These ‘master’ alignments contained the reference sequences as well as mitotic and flagellate sequences from the three species of interest. Separate “flagellate” and “mitotic” subalignments were then prepared for each species by only retaining flagellate or mitotic sequences from a given species, in addition to the common reference sequences. We quantified sequence conservation/divergence as a function of amino acid position in these subalignments using the AL2CO server,^73^ using normalized sum of pairs scoring (BLOSUM62 weighting) and otherwise default settings. The resulting conservation scores are normalized so that completely conserved positions return the same score regardless of the identity of the conserved amino acid; lower scores (including negative scores) correspond to less conservation. To assess differences in conservation between mitotic and flagellate sequences, the flagellate score was subtracted from the mitotic score at each amino acid position. The resulting difference score is close to zero when a position in the mitotic and flagellate sequences is equally conserved/diverged relative to the set of references sequences; it is positive when the mitotic sequence is less divergent, and negative when the mitotic sequence is more divergent. To identify the positions where the divergence of mitotic sequences was greater than flagellate sequences, the conservation score at each position was divided by the standard deviation of scores over all positions. We focused our subsequent analysis on especially divergent positions, which we defined as those where the relative divergence was greater than two standard deviations away from the mean (Figure 2A).

We used PyMol and a cryo-EM structure of αβ-tubulin in a microtubule (PDB: 6O2R)^82^ to assess if the especially divergent positions in mitotic tubulins were enriched near microtubule polymerization interfaces (Figures 2B, 2C, S2A, and S2B). To obtain the overall fraction of especially divergent positions per chain, the number of especially divergent positions in α- and β-tubulin was divided by the total number of amino acids. To calculate the proportion of divergent positions near lateral or longitudinal interfaces, we used distance based selections to identify the amino acids within a cutoff distance of a lateral or longitudinal lattice neighbor, and calculated the ratio of divergent to total positions within this subset.

#### Mitotic synchronies

To obtain a population of synchronized cells, we modified a previously published method^55^ to cause a heat-induced mitotic arrest. Briefly, the day before the synchrony, a lawn of *A. aerogenes* was collected in 10 ml of TrisMg (2 mM Tris + 10 mM MgSO4), pelleted, resuspended in 20 ml TrisMg. 10 ml of the bacterial solution were transferred into a 125 ml flask. 2–8×10^5^ amoebae were added to the flask and covered with foil, and the culture was incubated in a shaking water bath overnight (125 RPM, 30 °C). The morning of the synchrony, two additional lawns of *A. aerogenes* were collected, pelleted, and resuspended in 40 ml TrisMg. This solution was added to the flask with *Naegleria*, and allowed to shake for 3 minutes to thoroughly mix. This mixture was divided into 2 new (uncovered) flasks, one “control” and one “experimental,” and cell counts were taken with a hemocytometer. Cells were counted approximately every 20 min, and once the cells had doubled from their starting concentration, a sample was taken for quantitative real time PCR (qPCR) analysis (see next section), and the experimental flask was moved to a 38.5 +/−0.5 °C water bath. Cells were counted from each flask, and when the control flask had doubled again, another sample was taken from each flask for qPCR, and then the experimental flask was shifted back to 30 °C. Samples were taken from the experimental flask after shifting back to 30 °C to fix and stain cells for mitotic spindles.

#### Analysis of tubulin gene expression

Samples were collected from each flask prior to the temperature shift (pre-shift, control and experimental flasks), and again after incubation at 38 °C (or 30 °C for the control flask) but before shifting back to 30 °C. For each sample, 5 ml of cells were spun down at 1500 RCF at 4 °C for 5 min and the supernatant was discarded. The cell pellet was suspended in 1 ml TRIzol, vortexed, and promptly stored at −80 °C until RNA extractions. Cells were lysed using FastPrep homogenizer with bead beating in TRIzol. Lysate was cleaned up using a Zymo kit with on column DNase treatment, and RNA was eluted in 30 μl of kit-provided water. cDNA libraries were then generated using SuperScript IV First-Strand Synthesis System. cDNA, PowerSybr Green, and primers were mixed in triplicate in a MicroAmp Fast Optical 96-Well Reaction Plate with Barcode (Catalog #4346906) and sealed with an optical adhesive cover (Catalog #4360954). Genes targeted and primer sequences are presented in the key resources table. Samples were run on a StepOne Real-Time PCR machine and analyzed using StepOne software v2.3.

The fold change in mRNA abundance was determined from C_T_ values using the 2^−ΔΔCt^ method.^83^ Using this method, the flask that remained at 30 °C was a time-matched control for the experimental flask at the time point before the temperature shift, and the time point after the shift to 38 °C. *A Naegleria* G protein was used as the housekeeping gene to normalize the data, and a second housekeeping gene (GAPDH) was used to verify the results.

The microarray data in Figure 1D was originally acquired in Fritz-Laylin and Cande.^16^ Each biological replicate had been completed with 2 technical replicates, so the technical replicates were first averaged. Then, the mRNA abundance at the 0 min time point (before differentiation) and at the 80 min time point (after differentiation to flagellates) were compared for each of three biological replicates to calculate the fold change in mRNA abundance for mitotic and flagellate tubulins.

#### Growth assays with microtubule drugs

*Axenic Naegleria* amoebae (strain NEG-M) were diluted in M7 medium to a concentration of 2 × 10^5^ cells/ml and 500 μl of cell culture was added to each well of a 12-well tissue culture treated dish (Dot Scientific 667112). Drugs were diluted to a 2X concentration in M7 media, and 500 μl of each drug treatment was added to the corresponding well, and the plate was maintained at 28 °C without shaking for up to 40 h. Growth was measured at regular intervals by cell counting using a Moxi Z-series cell counter (Orflo Technologies) using Type S cassettes and diluent solution 75S (102 mM NaCl, 4 mM KCl, 11.25 mM Na2HPO4$H2O, 750 μM Na2EDTA, 7.5mM NaF). Cells were resuspended in the well by trituration with a p1000 pipette 10 times, and an aliquot was diluted 1:5 or 1:10 into 75S solution and mixed by trituration 3 more times immediately prior to counting. Bin precision was set to 3–26 μm, and the gated count method was used with gates set at 9.5 μm and 26 μm.

Drugs used were Benomyl, Colchicine, Nocodazole, Oryzalin, Paclitaxel, Plinabulin, and Vinblastine (see key resources table). Drugs were resuspended at stock concentrations below the solubility limit stated by the manufacturer. Colchicine was resuspended in water and all other drugs were resuspended in anhydrous DMSO (Sigma D2650). Drug solutions were stored in single-use aliquots at −20 °C until use.

#### Fluorescence microscopy

Immunofluorescence staining of amoebae and flagellates in Figure 1B was performed using an actin cytoskeleton fixation protocol modified from Velle and Fritz-Laylin.^15^ Cells were taken from an edge plate or from a sample of differentiated cells (see above), spun down at 1500 RCF for 90 sec, and cell pellets were resuspended in 1.5 ml 2 mM Tris. Cells were fixed in an equal volume of 2x fixative (50 mM sodium phosphate buffer pH 7.2, 125 mM sucrose, and 3.6% paraformaldehyde) for 15 minutes, then transferred to a 96 well glass-bottom plate coated with 0.1% poly(ethyleneimine) and allowed to settle for 15 min. Cells were rinsed twice in PEM (100 mM PIPES, 1 mM EGTA, 0.1 mM MgSO4; pH ~7.4) and permeabilized for 10 min in PEM + 0.1% NP-40 Alternative + 6.6 nM Alexa Fluor 488 Phalloidin (and 0.2x Tubulin Tracker Deep Red (prepared according to manufacturer instructions) columns 1, 2 and 4 only). Cells were rinsed twice in PEM, then blocked in PEMBALG (PEM + 1% BSA, 0.1% sodium azide, 100 mM lysine, and 0.5% cold water fish skin gelatin; pH 7.4) at room temperature for 1 h. Cells were then incubated in primary antibody (anti-α-tubulin mouse monoclonal antibody, clone DM1A) diluted to ~10 μg/ml in PEMBALG for 1 h. Cells were washed 3 times in PEMBALG, then incubated at room temperature for 1 h in Alexa Fluor 555 conjugated goat anti-mouse secondary antibody diluted to 2 μg/ml in PEMBALG, with 1x Tubulin Tracker Deep Red, ~66 nM Alexa Fluor 488 Phalloidin, and 1 μg/ml DAPI. Cells were then rinsed 4 times in PEM, and imaged the same day.

The *Naegleria* cells in Figure S3A were fixed in in an equal volume of 2x fixative (50 mM sodium phosphate buffer, 125 mM sucrose, and 3.6% paraformaldehyde) for 15 minutes, then transferred to plasma-cleaned coverslips coated with 0.1% poly(ethyleneimine) and allowed to settle for 15 min. Cells were spun to adhere to the plate at 1000 RCF for 3 min, then rinsed twice in PEM and permeabilized for 10 min at room temperature in PEM + 0.1% NP-40 Alternative. Samples were washed twice more in PEM and blocked for 1 h at room temperature in Detector Block (SeraCare) freshly prepared with 1% solids w/v. Cells were then incubated in primary antibodies targeting post-translational modifications (6–11B-1, 1:1000; YL1/2, 1:100; GT335, 1:100) diluted in Detector Block for 1 h, followed by incubation for 1 h with one of two primary antibodies against tubulin diluted in Detector Block (DM1A, 1:1000; YOL1/34, 1:100) chosen to be compatible with the host species of the antibody targeting the post-translational modification. Samples were washed 3 times in Detector Block then incubated for 1 h at room temperature with highly cross-adsorbed secondary antibodies (Alexa Fluor 488 conjugated goat anti-mouse, Alexa Fluor 647 conjugated goat anti-mouse; Alexa Fluor 647 conjugated goat anti-rat) at 1:500 dilution in Detector Block. Samples were washed 3 times with Detector Block and 3 times with PEM + 0.01% Triton-X 100, then mounted with Prolong Gold with DAPI and cured at room temperature overnight prior to imaging.

The cells in Figure S3B were incubated in 200 nM MitoTracker Red CMXRos for 10 min, then spun down and resuspended in 2 mM Tris three times. Cells were transferred to a 96 well glass bottom plate and imaged live. During live imaging, an equal volume of a 2x fixative and staining solution was added (100 mM sucrose, 100 mM sodium phosphate buffer, 4% PFA, 0.02% NP-40 Alternative, ~132 nM Alexa Fluor 488 Phalloidin, 2 μg/ml DAPI). Once the staining reached an optimal level (2–7 min after the addition of 2x fixative/staining solution), a single confocal slice was taken in a focal plane where DAPI-stained nuclei were in focus.

The porcine kidney cells (LLCPK1) in Figure S3A were fixed with either cold methanol (for use with the GT335 antibody) or paraformaldehyde/glutaraldehyde (for the 6–11B-1 and YL1/2 antibodies). For methanol fixation, cells adhered to coverslips were washed with 1X PBS and fixed for 10 minutes in a coplin jar containing 100% methanol at −20°C, followed by rehydration in PBS-tween-azide. For paraformaldehyde/glutaraldehyde fixation, cells were washed with 1X PBS and fixed for 10 minutes in 1X PBS containing 3.7% paraformaldehyde, 0.1% glutaraldehyde, and 0.5% Triton X-100, followed by rehydration in PBS-tween-azide. After rehydration cells from both fixation methods were then processed identically. Cells were incubated with primary antibody and 2% bovine serum albumin (BSA) in PBS-tween-azide for 1 h at 37 °C, washed in PBS-tween-azide, and incubated with secondary antibody and BSA for 45 min at room temperature in the dark. Stained cells were washed in PBS-tween-azide, mounted on ethanol-cleaned glass slides using DAPI fluoromount, and sealed with nail polish.

The images in Figures 1B, S3A, and S3B were taken on a Nikon Ti2 microscope equipped with a Plan Apo λ 100x oil objective (1.45 NA), a Crest spinning disk (50 μm), a Prime 95B CMOS camera, a Spectra III/Celesta light source for confocal illumination (at 50–100% power with excitation wavelengths of 405, 477, 546, and 638 nm) and a Sola light source for epifluorescence. The microscope was controlled through NIS Elements software, and images for Figure 1B were acquired as multi-channel z stacks with a step size of 200 nm and exposures of 200 ms (to image fluorescent phalloidin and tubulin antibody staining) or 500 ms (to image tubulin tracker staining). Images for Figure S3A had step sizes of 300 nm and exposure times of 400 ms for confocal; DAPI images were collected via epifluorescence at a single z-plane with the Sola light source at 90% power and an exposure time of 200 ms. Images for Figure S3B had exposure times of 250 ms (MitoTracker) or 1 s (DAPI).

Immunofluorescence staining in the remaining figures was optimized for microtubules and performed using amoeba from a fresh edge plate that had grown about half-way across the dish (or from a mitotic synchrony, detailed above). Cells were removed from the plate and added to approximately 3 mls of water in a conical tube, spun down in a clinical centrifuge at setting 7 for ~40 seconds and the supernatant removed leaving ~500 μl of water above the cell pellet. To this mixture an equal volume of freshly prepared 2X fixative solution consisting of 2 mM Tris pH 7.2; 125 mM sucrose; 10 mM NaCl, 2% paraformaldehyde was added and mixed gently. Cells were fixed for 10 min at room temperature. Cells were then placed on freshly coated coverslips and allowed to adhere for approximately 20–30 minutes. Coverslips were plasma cleaned and then coated with 0.1% poly(ethyleneimine). After cells were adhered to the coverslips, they were rinsed 3 times with 1 ml of PEM and then permeabilized with 0.1% NP-40 Alternative for 10 minutes. Cells were blocked in PEM-BALG for one hour or overnight and then incubated with primary antibody for 1 hour at 37 °C or at room temperature overnight. Coverslips were rinsed in PBS containing 0.1% Tween and 0.02% sodium azide and incubated with Dylight-488 labeled anti-mouse secondary antibodies (Invitrogen) according to the manufacturers’ recommended protocol. Finally, coverslips were washed in PEM supplemented with 0.01% Triton-X-100 for 5 minutes before mounting on clean slides using DAPI Fluoromount G (Southern Biotech) or Prolong Gold.

These cells were imaged on a Nikon Ti-E microscope with a CSU-X1 Yokogawa spinning-disk confocal scan head (PerkinElmer, Wellesley, MA), an Andor iXon+ electron-multiplying charge-coupled device camera (Andor), using a 100X/1.4 NA objective lens. Z-step size was set at 0.2 μm.

Laser powers and exposures were chosen to ensure that the fluorescent signal would not be saturated and were adjusted depending on the fluorescent signal. For imaging microtubules with a Dylight 488 labeled secondary antibody, images were acquired using a 488 nm laser at 10.2% power; for imaging DNA, the 405 nm laser was used at 40.2% power.

#### Transmission Electron Microscopy

Cells were fixed overnight at 4 °C in 2.5% glutaraldehyde + 100 mM sodium cacodylate, then rinsed and stored in 100 mM sodium cacodylate overnight. Samples were then rinsed in 100 mM sodium cacodylate buffer, pH 7.4, three times for 10 minutes per wash. Cells were post fixed in 1% aqueous osmium tetroxide (Electron Microscopy Sciences) in 100 mM sodium cacodylate buffer overnight at 4 °C. Cells were then rinsed twice in water for 10 min per wash, before en bloc staining with 1% uranyl acetate (Electron Microscopy Sciences) in water for 1 hour at room temperature. Cells were rinsed 3 times in water, for 10 min per wash. Cells were then subjected to a graded ethanol dehydration series as follows with 15 min washes at each of the following ethanol concentrations: 50%, 70%, 80%, 90%, 95%, followed by two ten minute washes in 100% ethanol. Cells were quickly rinsed in propylene oxide, then infiltrated with 50% resin (Araldite 502/Embed-12, Electron Microscopy Sciences) and propylene oxide overnight. Cells were then incubated for 6–12 hours in each of the following resin concentrations: 70%, 85%, 95%, and 100% followed by embedding in 100% resin at 60 °C for 4 days. ~70 nm thin sections were cut using an RMC PowerTime XL Ultramicrotome with a Diatome diamond knife, and were transferred to copper grids. Sections were post stained with 1% uranyl acetate for 6 min, and lead citrate for 2 min. Images were taken using a JEOL JEM-200CX transmission electron microscope.

### QUANTIFICATION AND STATISTICAL ANALYSIS

The sample size for each relevant figure panel is included in the figure legends and also summarized in Table S2. The relevant measures of the center and dispersion of distributions are described in the figure legends. No statistical analysis was performed. Specific approaches for quantification are described below.

#### Deconvolution and 3D reconstruction

Z stacks captured using a spinning disk confocal microscope were digitally deconvolved using Autoquant X3 software. The default 3D deconvolution settings for spinning disk confocal data were used with “expert recommended settings,” and 40 iterations. The deconvolved images were then processed in Fiji^74^ to set the scaling, and to remove the mitochondria prior to 3D rendering, as the intensely-stained mitochondria made it difficult to observe the DNA in the nucleus. The resulting deconvolved image stacks were used to generate 3D surface renderings in UCSF ChimeraX software.^75^

#### Analysis of spindle morphology

Spindle length and width measurements were assessed using the raw confocal (not deconvolved) datasets, and were only measured for spindles lying parallel to the plane of the coverslip. Length was measured by drawing a line in Fiji using the straight line tool, and measuring from the end of one pole to the opposite pole. For spindles in prophase where the poles are unclear, the longest axis was measured. In cases where the spindle bent during telophase (e.g. Figure 3A, Anaphase/Telophase), the segmented line tool was used to follow the length of the spindle more accurately. Spindle width was measured using only the straight line tool, and was assessed at the approximate midpoint of the spindle between the two poles. These length and width values were separated by spindle stage, and were plotted using GraphPad Prism 8 software. The number of experimental and technical replicates for all graphs are listed in Table S2.

The number of bundles and the distance between bundles were calculated from confocal Z-stacks of metaphase spindles lying perpendicular to the coverslip. Bundle number was assessed in each plane going through the bundle for 8 representative spindles (Figure 4B), and the maximum number of bundles present at the midplane was calculated for additional metaphase spindles. To determine the average distance between bundles, a frame that represented the spindle midplane was used, and the center of each bundle was selected using the multi-point tool in Fiji. The coordinates of each bundle center were used to determine the distance from each bundle to its two nearest neighboring bundles.

Line scan analysis (Figures 4D, S4G, and S4H) was completed using confocal images of spindles that were oriented parallel to the coverslip. Image stacks were first transformed into sum intensity projections in Fiji. Then, the line width was matched to the width of the spindle, and a line (or segmented line in the case of bent anaphase/telophase spindles) was drawn to include the entire spindle length, with a short length of background at each end. The “plot profile” tool in Fiji was then used to extract the average pixel intensity along the line for tubulin and DNA staining. These values were normalized to the average intensity of an area of the cell adjacent to the spindle, which was set to 1. The spindle lengths were also normalized such that “0” represents the midpoint of the spindle. To determine the relative quantity of DNA and tubulin in these spindles (Figure 4E), the area under the linescan-generated curves was calculated using GraphPad Prism 8 software, using a baseline level of 1.

#### Analysis of spindle twist

To characterize the shape of microtubule bundles, we manually tracked individual bundles of vertically oriented spindles, and horizontally oriented spindles whose image stacks were first transformed into vertical (end-on) orientation, using the Multipoint tool in Fiji. As microtubule bundles appear as spots in a spindle cross-section, each point was placed at the center of the signal and its x,y,z coordinates were saved. Moving up and down through the z-stack helped to determine this point. Each bundle was tracked through all z-planes where it was visible. Positions of the spindle poles were also determined, as the spots in the center of the end points of all bundles in the plane beyond the bundle ends. Coordinates of bundles and poles were transformed so that both poles are on the z-axis.

To describe the shape of a microtubule bundle, we fit a plane to the points representing the bundle. Subsequently, we fit a circle that lies in this plane to the same points. These fits were used to calculate the curvature and twist of the bundle as follows: (i) The curvature is calculated as one over the radius, and (ii) the twist is calculated as the angle between the plane and the z-axis divided by the mean distance of these points from the z-axis. Bundle length was calculated as the length of the projection of the bundle trace onto the pole-to-pole axis. For detailed descriptions of this method, see Ivec et al.^76^

## Supplementary Material

1

2

3

4

Data S1

Data S3

Data S4

Data S5

Data S6

Data S2

## Figures and Tables

**KEY RESOURCES TABLE:** 

REAGENT or RESOURCE	SOURCE	IDENTIFIER

Antibodies		

Mouse monoclonal anti-α-tubulin antibody (DM1A)	Sigma Aldrich	Cat#T6199; RRID: AB_477583
Rat monoclonal anti-α-tubulin antibody (YOL1/34)	Abcam	Cat#ab6161; RRID: AB_305329
Mouse monoclonal anti-acetylated α-tubulin antibody (6-11B-1)	Sigma Aldrich	Cat#T7451; RRID: AB_609894
Rat monoclonal anti-α-tubulin antibody (YL1/2)	Abcam	Cat#ab6160; RRID: AB_305328
Mouse monoclonal anti-polyglutamylation antibody	AdipoGen	Cat#AG-20B-0020; RRID: AB_2490210
AF555 goat anti-mouse highly cross-adsorbed secondary antibody	Thermo Fisher	Cat#A21424; RRID: AB_141780
AF488 Goat anti-mouse highly cross-adsorbed secondary antibody	Thermo Fisher	Cat#A32723; RRID: AB_2633275
AF647 Goat anti-mouse highly cross-adsorbed secondary antibody	Thermo Fisher	Cat#A21236; RRID: AB_2535805
AF647 Goat anti-rat highly cross-adsorbed secondary antibody	Thermo Fisher	Cat#A48265; RRID: AB_2895299

Bacterial and virus strains		

*Aerobacter aerogenes*	Chandler Fulton	N/A

Chemicals, peptides, and recombinant proteins		

Difco Antibiotic Medium 3	Fisher	Cat#DF0243-17-8
Gibco Bacto Peptone	Fisher	Cat#DF0118-17-0
Gibco Yeast Extract	Fisher	Cat#B11929
TRIZol Reagent	Thermo Fisher	Cat#15596026
Benomyl	Sigma Aldrich	Cat#45339
Colchicine	Sigma Aldrich	Cat#C9754
Nocodazole	Sigma Aldrich	Cat#M1404
Oryzalin	Sigma Aldrich	Cat#36182
Paclitaxel	Sigma Aldrich	Cat#T7402
Plinabulin	Sigma Aldrich	Cat#ADV947322154
Vinblastine	Sigma Aldrich	Cat#V1377
DMSO (anhydrous)	Sigma Aldrich	Cat#D2650
NP-40 Alternative	Sigma Aldrich	Cat#492016
Detector Block	SeraCare	Cat#5920-0004
Triton-X 100	Promega	Cat#H5142
cold water fish skin gelatin	Sigma Aldrich	Cat#G7765
Tubulin Tracker Deep Red	Thermo Fisher	Cat#T34077
MitoTracker Red CMXRos	Thermo Fisher	Cat#M7512
AF488 Phalloidin	Thermo Fisher	Cat#A12379
Prolong Gold mounting medium with DAPI	Thermo Fisher	Cat#P36935

Critical commercial assays		

Power SYBR Green qPCR Master Mix	Thermo Fisher	Cat#4368706
SuperScript IV First-Strand Synthesis kit	Thermo Fisher	Cat#18091200

Experimental models: Organisms/strains		

*Naegleria gruberi:* NEG: wildtype	Chandler Fulton	ATCC 30223
*Naegleria gruberi:* NEG-M: wildtype	Chandler Fulton	ATCC 30224

Oligonucleotides		

*N. gruberi* GAPDH (JGI ID: 53883) qPCR primers: Forward 5’-TGGC TCCAATTGCTGCTGTTT-3’ and reverse 5’-CCTTAGCAGCACCAG TTGAAGA-3’	This work	N/A
*N. gruberi* G Protein (JGI ID: 77952) qPCR primers: Forward 5’-ACGGTTGGGTCACTTGTTTGTCC-3’ and reverse 5’-GAGCGTGACCAGTGAGGGATC-3’	This work	N/A
*N. gruberi* mitotic α-tubulin (JGI ID: 58607) qPCR primers: Forward 5’-GGTCCTTGATGTGTGCCGAAC-3’ and reverse 5’-TTAGCAGCATCTTCACGACCAGT-3’	This work	N/A
*N. gruberi* mitotic α-tubulin (JGI ID: 55745) qPCR primers: Forward 5’-CACACACAAAATGAGAGAAGTCGTC-3’ and reverse 5’-TTCCATGTTCAGCACAGAATAATTC-3’	This work	N/A
*N. gruberi* mitotic β-tubulin (JGI ID: 55748) qPCR primers: Forward 5’-AACCAACACTGCTTCTCCACTCG-3’ and reverse 5’-TCTGGACGGAATAATTGACCTTGG-3’	This work	N/A
*N. gruberi* mitotic β-tubulin (JGI ID: 55900) qPCR primers: Forward 5’-GGTTGCTGGTGTCATGTCTGGTG-3’ and reverse 5’-GCAGCCAAAGGAGCAGAACCAA-3’	This work	N/A

Software and algorithms		

PhyloTOL	68	N/A
PASTA multiple sequence aligner	69	N/A
IQ-Tree 2 v.1.16.2	70	RRID: SCR_017254
ITOL v4	71	RRID: SCR_018174
ClustalOmega	72	RRID: SCR_001591
AL2CO	73	N/A
PyMOL v2.4.1	Schrödinger, LLC	RRID: SCR_000305
NIS Elements with Advanced Research Package	Nikon Instruments	RRID: SCR_014329
Autoquant X3 vX3.1.3	Media Cybernetics	RRID: SCR_002465
Fiji	74	RRID: SCR_002285
ChimeraX	75	RRID: SCR_015872
GraphPad Prism v8	GraphPad	RRID: SCR_002798
Custom scripts to analyze spindle twist	76	N/A
StepOne v2.3	Thermo Fisher	RRID: SCR_014281

## Highlights

- *Naegleria* expresses evolutionarily divergent α- and β-tubulins only during mitosis
- Mitotic tubulins differ at key lateral and longitudinal microtubule interfaces
- The mitotic spindle is a ring of regularly spaced microtubule bundles that twists
- The length and number of microtubule bundles increase as mitosis proceeds
