# Supplementary material for: Naegleria’s mitotic spindles are built from unique tubulins and highlight core spindle features
Source: Curr Biol. Author manuscript; Available in PMC 2022 Apr 25. (PMC9036621; doi:10.1016/j.cub.2022.01.034)
Supplement: 1 [file NIHMS1779381-supplement-1.pdf]

Current Biology, Volume *32*

Supplemental Information

***Naegleria*'s mitotic spindles are built from unique tubulins and highlight core spindle features**

Katrina B. Velle, Andrew S. Kennard, Monika Trupinić, Arian Ivec, Andrew J.M. Swafford, Emily Nolton, Luke M. Rice, Iva M. Tolić, Lillian K. Fritz-Laylin, and Patricia Wadsworth

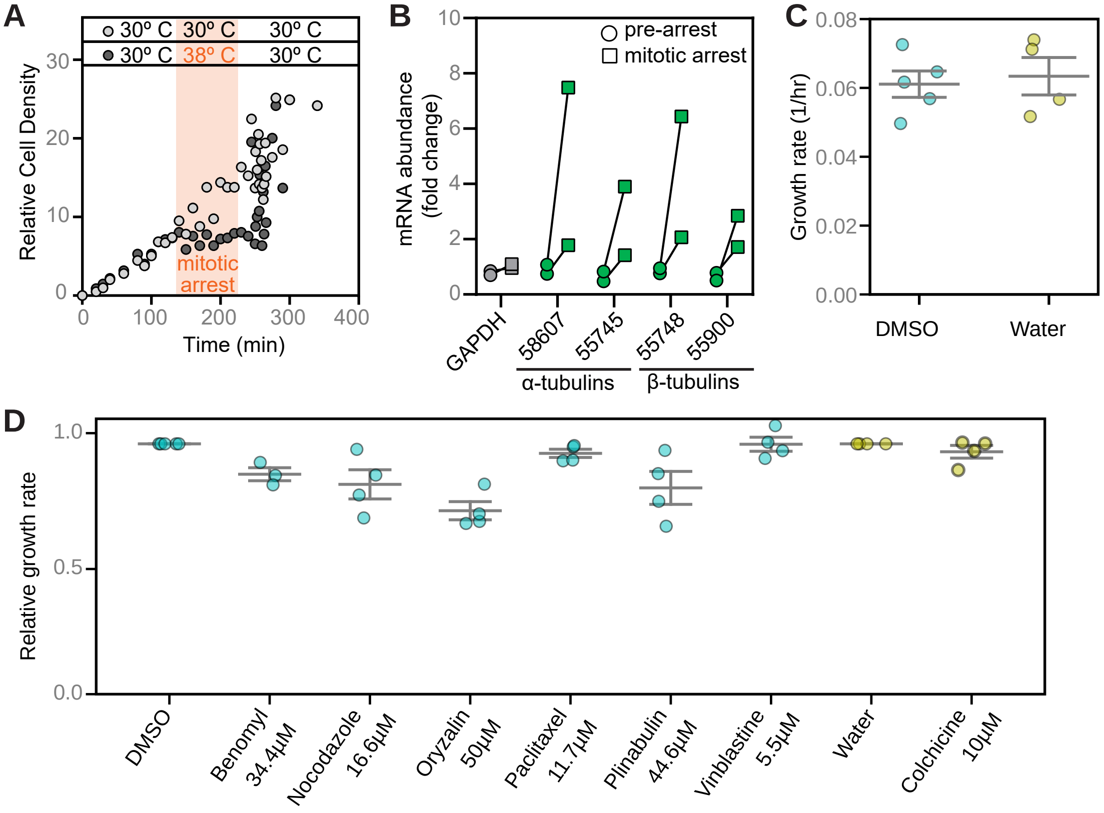

E

α-tubulins

					flagellate α-tubulins (heteroloboseans)				mitotic α-tubulins (heteroloboseans)				
	H. sapiens (ENSP-00000336799)	*C. reinhardtii* (186023)	*S. cerevisiae* (288cYML085C)	*S. cerevisiae* (288cYML124C)	*N. gruberi* (53284)	*N. gruberi* (56236)	*A. kona* (12591)	*N. fowleri* (3292)	*N. gruberi* (58607)	*N. gruberi* (55745)	*A. kona* (12838)	*N. fowleri* (5134)	*N. fowleri* (7486)
N. fowleri (7486)	57	60	55	55	60	61	60	61	71	88	67	73	100
N. fowleri (5134)	55	58	53	53	57	57	57	57	83	71	64	100	
A. kona (12838)	63	65	60	60	63	65	65	64	65	66	100		
N. gruberi (55745)	58	62	56	55	61	62	63	62	73	100			
N. gruberi (58607)	57	59	54	54	58	59	59	59	100				
N. fowleri (3292)	85	92	68	67	88	98	97	100					
A. kona (12591)	84	92	69	67	87	97	100						
N. gruberi (56236)	85	92	68	67	88	100							
N. gruberi (53284)	79	86	68	67	100								
S. cerevisiae (288cYML124C)	72	69	88	100									
S. cerevisiae (288cYML085C)	74	69	100										
C. reinhardtii (186023)	86	100											
H. sapiens (ENSP00000336799)	100												

β-tubulins

				flagellate β-tubulins (heteroloboseans)			mitotic β-tubulins (heteroloboseans)				
	H. sapiens (ENSP-00000217133)	*C. reinhardtii* (129876)	*S. cerevisiae* (288cYFL037W)	*N. gruberi* (83350)	*A. kona* (08655)	*N. fowleri* (5358)	*N. gruberi* (55748)	*N. gruberi* (55900)	*A. kona* (11976)	*N. fowleri* (5966)	*N. fowleri* (3784)
N. fowleri (3784)	57	63	62	63	63	63	70	88	67	71	100
N. fowleri (5966)	56	59	57	60	60	60	86	71	62	100	
A. kona (11976)	60	66	64	67	67	67	62	65	100		
N. gruberi (55900)	58	63	62	63	64	64	71	100			
N. gruberi (55748)	57	60	58	61	61	60	100				
N. fowleri (5358)	74	90	72	97	96	100					
A. kona (08655)	73	91	72	96	100						
N. gruberi (83350)	75	91	72	100							
S. cerevisiae (288cYFL037W)	65	70	100								
C. reinhardtii (129876)	73	100									
H. sapiens (ENSP00000217133)	100										

% Identity

0 — 100

Figure S1. The *Naegleria* tubulins expressed during mitosis are divergent. Related to Figure 1. (**A**) To enhance the percent of mitotic cells, a population of cells grown at 30 °C was shifted to 38 °C (dark gray points, orange panel) to induce a mitotic arrest. After shifting cells back to 30 °C, cells divide synchronously, quickly catching up to the density of cells in a control flask left at 30 °C (light gray points). Each point represents the relative cell density of a flask at a given time point (for 9-18 timepoints per flask), for 5 independent synchrony experiments. (**B**) Samples of cells from a mitotic synchrony experiment were subjected to qPCR analysis to determine mRNA levels from housekeeping genes (GAPDH, gray, and G protein, used for normalization), and mitotic α and tubulins (green). The fold change in mRNA abundance before (circles) or after (squares) the 38 °C mitotic arrest was calculated relative to a control flask kept at 30 °C. Each point represents one biological replicate consisting of 3 technical replicates. (**C**) Growth rates of *Naegleria gruberi* axenic strain NEG-M in growth media supplemented with DMSO or water. Each data point is an independent biological replicate from a different day. Mean +/- Standard Error (SE) is shown in gray lines. (**D**) Relative growth rates of NEG-M in growth media supplemented with the specified concentration of each inhibitor. Growth rates are normalized to the appropriate vehicle control, indicated by the color of the dot. Mean +/- SE is shown with gray lines. (**E**) The percent identities of α (top) and (bottom) tubulin sequences were calculated for different tubulins in multiple species. Purple indicates lower % ID, while gray indicates a higher % ID.

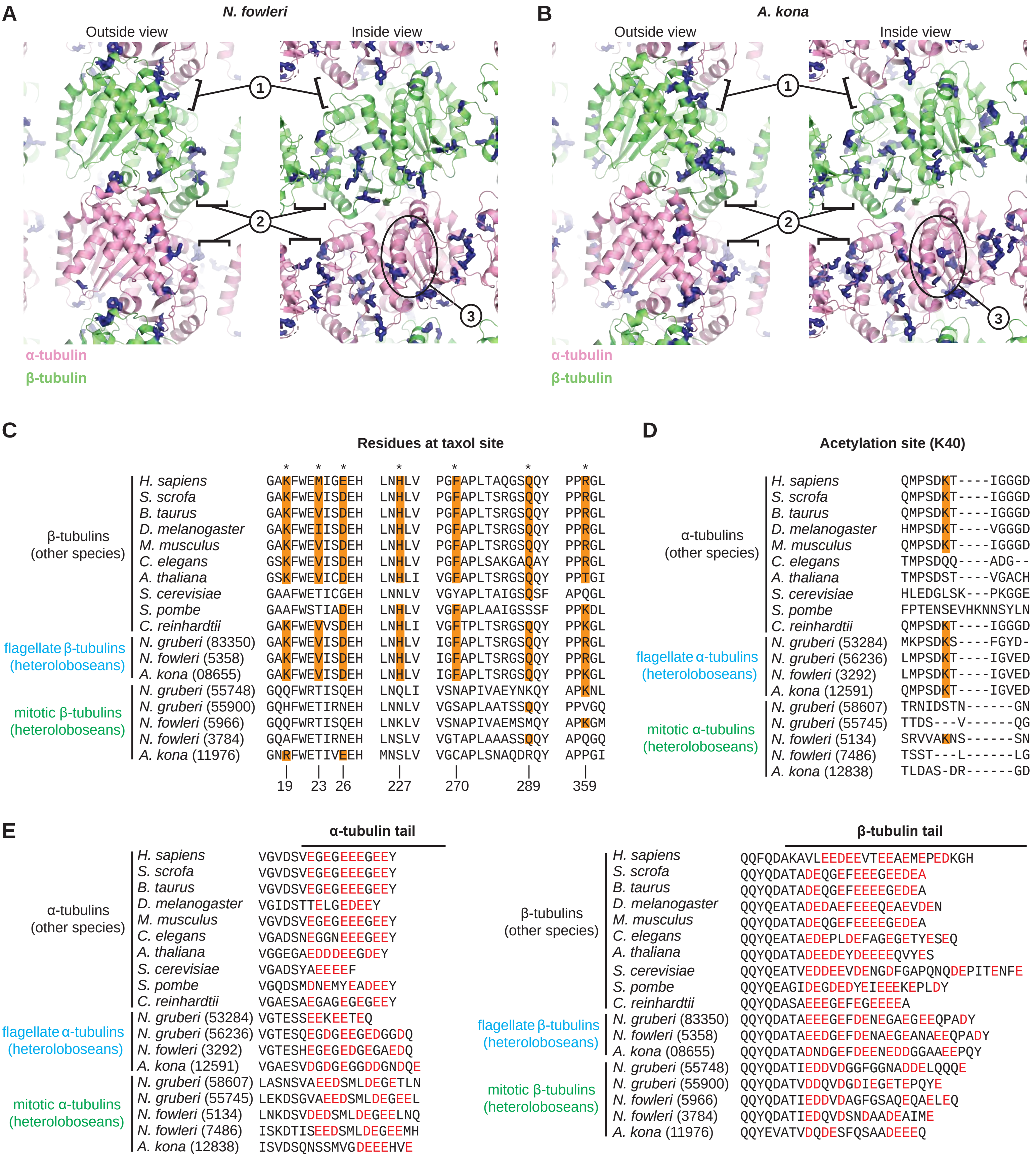
A
N. fowleri
Outside view
Inside view
1
2
3
α-tubulin
β-tubulin
B
A. kona
Outside view
Inside view
1
2
3
α-tubulin
β-tubulin
C
Residues at taxol site
β-tubulins (other species)
H. sapiens GAKFWEMIGEEH LNHLV PGFAPLTAQGSQQY PPRGL
S. scrofa GAKFWEVISDEH LNHLV PGFAPLTSRGSQQY PPRGL
B. taurus GAKFWEVISDEH LNHLV PGFAPLTSRGSQQY PPRGL
D. melanogaster GAKFWEIISDEH LNHLV PGFAPLTSRGSQQY PPRGL
M. musculus GAKFWEVISDEH LNHLV PGFAPLTSRGSQQY PPRGL
C. elegans GSKFWEVISDEH LNHLV PGFAPLSAKGAQAY PPRGL
A. thaliana GSKFWEVICDEH LNHLI VGFAPLTSRGSQQY PPTGI
S. cerevisiae GAAFWETICGEH LNNLV VGYAPLTAIGSQSF APQGL
S. pombe GAAFWSTIADEH LNHLV VGFAPLAAIGSSSF PPKDL
C. reinhardtii GAKFWEVVSDEH LNHLI VGFTPLTSRGSQQY PPKGL
flagellate β-tubulins (heterolobоseans)
N. gruberi (83350) GAKFWEVISDEH LNHLV IGFAPLTSRGSQQY PPRGL
N. fowleri (5358) GAKFWEVISDEH LNHLV IGFAPLTSRGSQQY PPRGL
A. kona (08655) GAKFWEVISDEH LNHLV IGFAPLTSRGSQQY PPKGL
mitotic β-tubulins (heterolobоseans)
N. gruberi (55748) GQQFWRTISQEH LNQLI VSNAPIVAEYNKQY APKNL
N. gruberi (55900) GQHFWETIRNEH LNNLV VGSAPLAATSSQQY PPVGQ
N. fowleri (5966) GQQFWRTISQEH LNKLV VSNAPIVAEMSMQY APKGM
N. fowleri (3784) GQAFWETIRNEH LNSLV VGTAPLAAASSQQY APQGQ
A. kona (11976) GNRFWETIVEEH MNSLV VGCAPLSNAQDRQY APPGI
19 23 26 227 270 289 359
D
Acetylation site (K40)
α-tubulins (other species)
H. sapiens QMPSDKT----IGGGD
S. scrofa QMPSDKT----IGGGD
B. taurus QMPSDKT----IGGGD
D. melanogaster HMPSDKT----VGGGD
M. musculus QMPSDKT----IGGGD
C. elegans TMPSDQQ----ADG--
A. thaliana TMPSDST----VGACH
S. cerevisiae HLEDGLSK---PKGGE
S. pombe FPTENSEVHKNNSYLN
C. reinhardtii QMPSDKT----IGGGD
flagellate α-tubulins (heterolobоseans)
N. gruberi (53284) MKPSDKS----FGYD-
N. gruberi (56236) LMPSDKT----IGVED
N. fowleri (3292) LMPSDKT----IGVED
A. kona (12591) QMPSDKT----IGVED
mitotic α-tubulins (heterolobоseans)
N. gruberi (58607) TRNIDSTN------GN
N. gruberi (55745) TTDS---V------QG
N. fowleri (5134) SRVVAKNS------SN
N. fowleri (7486) TSST---L------LG
A. kona (12838) TLDAS-DR------GD
E
α-tubulin tail
α-tubulins (other species)
H. sapiens VGVDSVEGEGEEEGEEY
S. scrofa VGVDSVEGEGEEEGEEY
B. taurus VGVDSVEGEGEEEGEEY
D. melanogaster VGIDSTTELGEDEEY
M. musculus VGVDSVEGEGEEEGEEY
C. elegans VGADSNEGGNEEEGEEY
A. thaliana VGGEGAEDDDEEGDEY
S. cerevisiae VGADSYAEEEEF
S. pombe VGQDSMDNEMYEADEEY
C. reinhardtii VGAESAEGAGEGEGEEY
flagellate α-tubulins (heterolobоseans)
N. gruberi (53284) VGTESSEEKEETEQ
N. gruberi (56236) VGTESQEGDGEEGEDGGDQ
N. fowleri (3292) VGTESHEGEGEDGEGAEDQ
A. kona (12591) VGAESVDGDGEGGDDGNDQE
mitotic α-tubulins (heterolobоseans)
N. gruberi (58607) LASNSVAEEDSMLDEGETLN
N. gruberi (55745) LEKDSGVAEEDSMLDEGEEL
N. fowleri (5134) LNKDSVDEDSMLDEGEELNQ
N. fowleri (7486) ISKDTISEEDSMLDEGEEMH
A. kona (12838) ISVDSQNSSMVGDEEEHVE
β-tubulin tail
β-tubulins (other species)
H. sapiens QQFQDAKAVLEEDEEVTEEAEMEPEDKGH
S. scrofa QQYQDATADEQGEFEEEGEEDEA
B. taurus QQYQDATADEQGEFEEEGEEDEA
D. melanogaster QQYQEATADEDAEFEEEQEAEVDEN
M. musculus QQYQDATADEQGEFEEEEGEDEA
C. elegans QQYQEATAEDEPLDEFAGEGETYESEQ
A. thaliana QQYQDATADEEDEYDEEEEQVYES
S. cerevisiae QQYQEATVEDDEEVDENGDFGAPQNQDEPITENFE
S. pombe QQYQEAGIDEGDEDYEIEEEKEPLDY
C. reinhardtii QQYQDASAEEEGEFEGEEEEA
flagellate β-tubulins (heterolobоseans)
N. gruberi (83350) QQYQDATAEEEGEFDENEGAEGEEQPADY
N. fowleri (5358) QQYQDATAEEDGEFDENAEGEANAEEQPADY
A. kona (08655) QQYQDATADNDGEFDEENEDDGGAAEEPQY
mitotic β-tubulins (heterolobоseans)
N. gruberi (55748) QQYQDATIEDDVDGGFGGNADDELQQQE
N. gruberi (55900) QQYQDATVDDQVDGDIEGETEPQYE
N. fowleri (5966) QQYQDATIEDDVDAGFGSAQEQAELEQ
N. fowleri (3784) QQYQDATIEDQVDSNDAADEAIME
A. kona (11976) QQYEVATVDQDESFQSAADEEEQ

Figure S2. Structural context of the sites with increased divergence in the mitotic tubulins. Related to Figure 2. (**A**) Side-chain positions for the *N. fowleri* amino acids identified in **Figure 2A** are represented as sticks (blue) on a model of αβ-tubulin in the microtubule lattice (α-tubulin: pink, β-tubulin: lime). 'Outside' and 'Inside' views of the lattice are shown, and longitudinal (labeled 1) and lateral (labeled 2) microtubule lattice contacts are indicated, as is the luminal (internal) surface of α-tubulin (labeled 3). (**B**) As in A, but for the side-chain positions for the *A. kona* amino acids identified in **Figure 2A**. (**C**) Key differences in taxol binding residues between mitotic and flagellate sequences: flagellate β-tubulins have generally conserved residues implicated in taxol binding (highlighted orange), but the mitotic β-tubulins have not. (**D**) Flagellate α-tubulins have generally conserved a K40-equivalent residue (highlighted in orange); this position is subject to acetylation in more commonly studied organisms. Mitotic α-tubulins have diverged in this region. (**E**) The mitotic α-tubulins are notably lacking the C-terminal tyrosine (Y) that is subject to detyrosination/retyrosination in more commonly studied organisms. Besides that, there are few obvious differences in the length or overall charge (negatively charged amino acids are colored red) between mitotic and flagellate α- or β-tubulins.

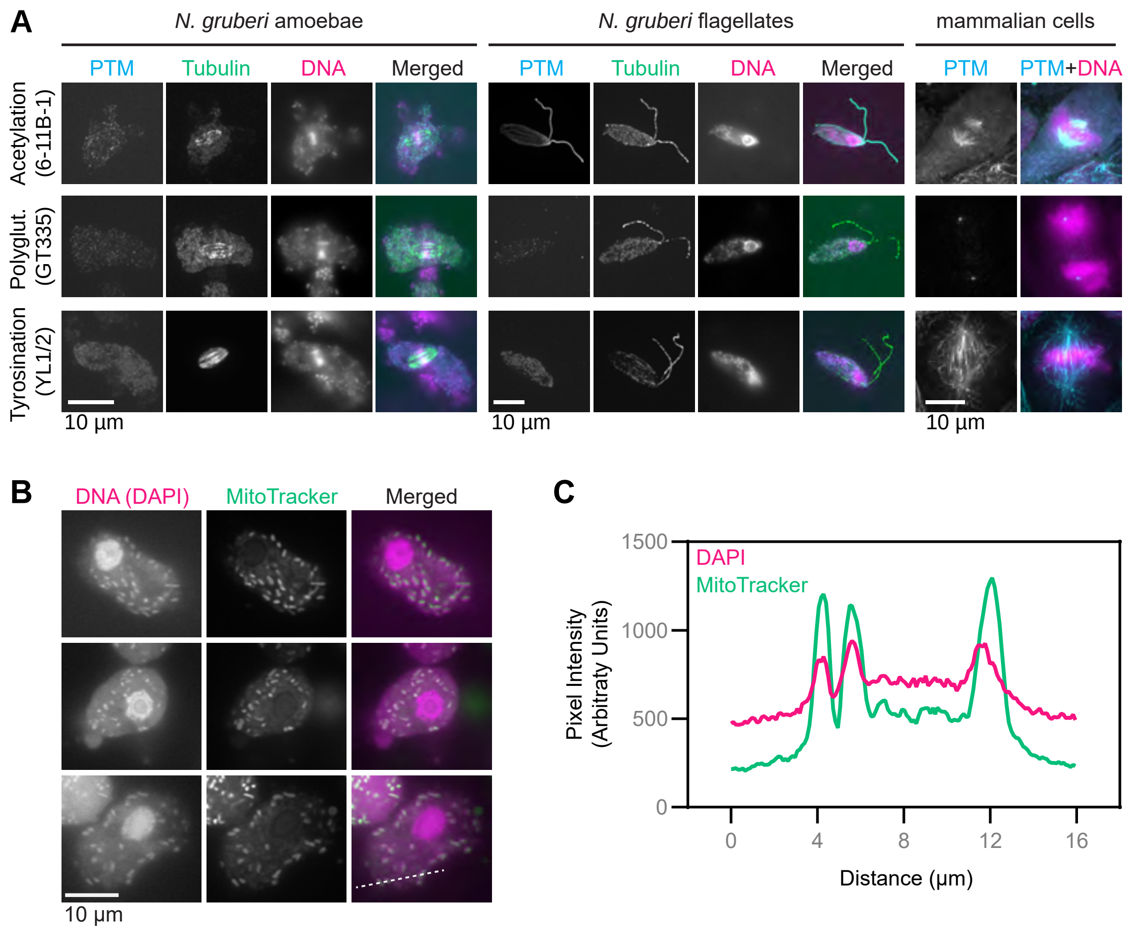

Figure S3. *Naegleria's* mitotic tubulins do not have conventional post-translational modifications. Related to Figure 2 and Figure 3. (**A**) Maximum intensity projections of *Naegleria* amoebae, flagellates, and porcine kidney cells (LLCPK1) immunostained for post-translational tubulin modifications. *Naegleria* cells were sequentially stained first with antibodies for the specified post-translational modifications, followed by a general tubulin antibody to identify spindles or flagella. Each row is labeled with the type of post-translational modification stained for, with the specific antibody clone used in parentheses. Staining of the spindle is not observed with any of the antibodies against acetylated, polyglutamylated, or tyrosinated tubulin, while flagellates stain robustly for acetylated tubulin, but not polyglutamylation or tyrosination. Porcine kidney cells (LLCPK1) demonstrate staining with all three antibodies as a positive control: Spindle microtubules are stained with antibodies against acetylated and tyrosinated tubulin, and centrosomes are stained with antibodies against polyglutamylated tubulin. (**B**) *Naegleria*'s mitochondria stain with DAPI. Amoebae were incubated with MitoTracker™ Red CMXRos to stain mitochondria, and were then fixed and stained with DAPI to label DNA. One confocal z plane from each of three representative cells are shown. (**C**) The dashed line in panel B (drawn through three mitochondria), was used to generate a pixel intensity plot for DAPI and MitoTracker staining.

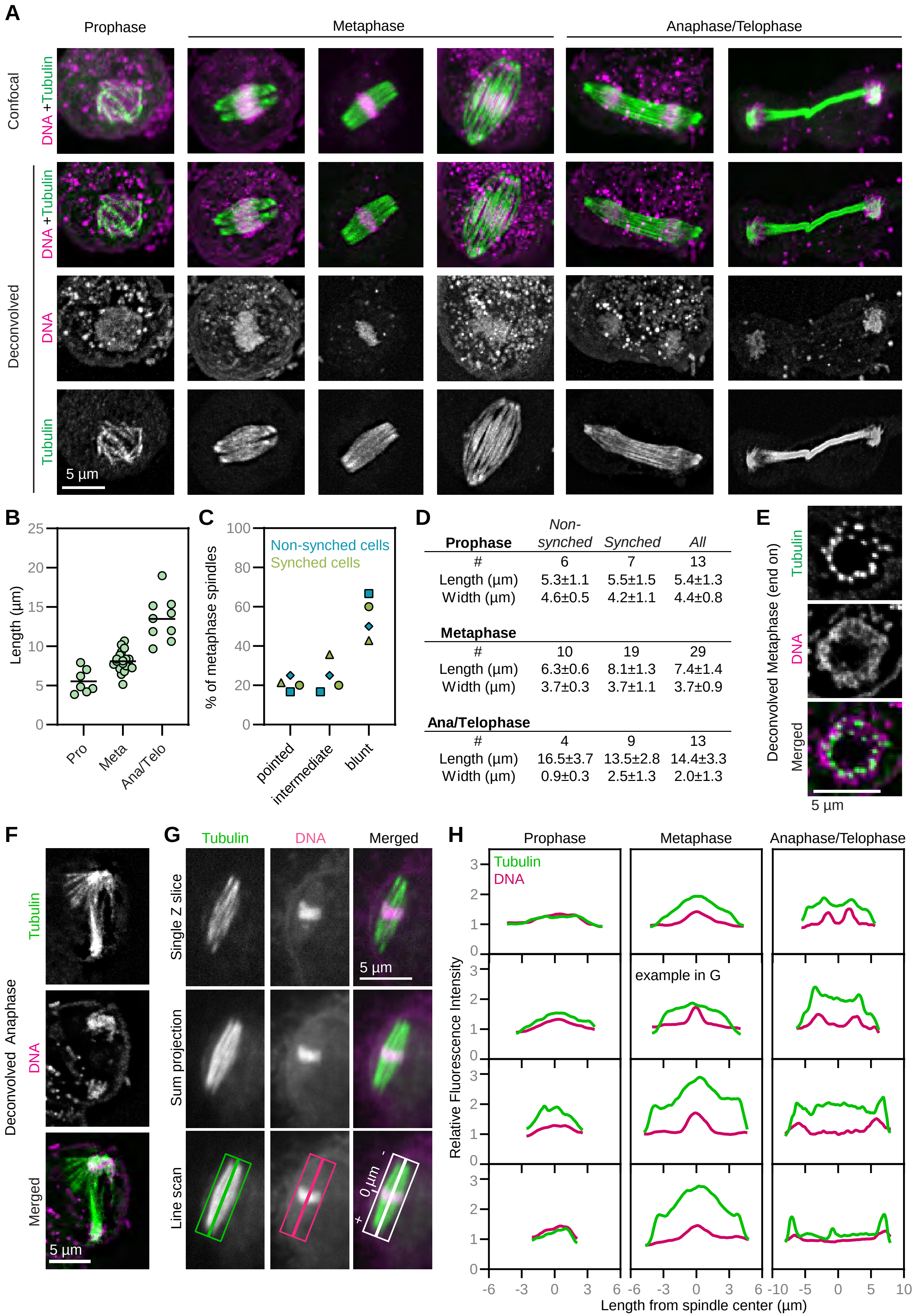
A
Prophase
Metaphase
Anaphase/Telophase
Confocal
DNA + Tubulin
Deconvolved
DNA + Tubulin
DNA
Tubulin
5 µm
B
Length (µm)
0
5
10
15
20
25
Pro
Meta
Ana/Telo
C
% of metaphase spindles
0
20
40
60
80
100
Non-synched cells
Synched cells
pointed
intermediate
blunt
D
Prophase | Non-synched | Synched | All
| 6 | 7 | 13
Length (µm) | 5.3±1.1 | 5.5±1.5 | 5.4±1.3
Width (µm) | 4.6±0.5 | 4.2±1.1 | 4.4±0.8
Metaphase
| 10 | 19 | 29
Length (µm) | 6.3±0.6 | 8.1±1.3 | 7.4±1.4
Width (µm) | 3.7±0.3 | 3.7±1.1 | 3.7±0.9
Ana/Telophase
| 4 | 9 | 13
Length (µm) | 16.5±3.7 | 13.5±2.8 | 14.4±3.3
Width (µm) | 0.9±0.3 | 2.5±1.3 | 2.0±1.3
E
Deconvolved Metaphase (end on)
Tubulin
DNA
Merged
5 µm
F
Deconvolved Anaphase
Tubulin
DNA
Merged
5 µm
G
Tubulin
DNA
Merged
Single Z slice
Sum projection
Line scan
5 µm
0 µm
-
+
H
Prophase
Metaphase
Anaphase/Telophase
Tubulin
DNA
example in G
Relative Fluorescence Intensity
0
1
2
3
-6
-3
0
3
6
-10
-5
5
10
Length from spindle center (µm)

Figure S4. Mitotically synchronized amoebae have indistinguishable spindle architecture from unsynchronized cells. Related to Figure 3. (**A**) Amoebae were synchronized, then fixed and stained with antibodies (anti-alpha tubulin clone DM1A, green) to detect microtubules, and DAPI to label DNA (magenta). Cells were imaged using confocal microscopy (top row), and the images were deconvolved using Autoquant software (bottom rows). All images are maximum intensity projections. Cells were classified as prophase, metaphase, or anaphase/telophase. (**B**) Cells were treated as in A, and confocal images were used to quantify the maximum spindle length. Each point represents one mitotic spindle, and lines indicate the averages. Measurements were taken from 35 cells encompassed by two experimental replicates. (**C**) Metaphase spindles from synchronized experiments (green data points) and non-synchronized experiments (blue data points) were scored as having pointed ends (see the first metaphase cell in **Figure 3A** as an example), blunt ends (see the second metaphase cell in panel A of this figure), or an intermediate phenotype that did not clearly fall into the pointed or blunt category (see third metaphase cell in panel A). Each symbol represents one experimental replicate, coordinated by shape. Data are from 4 experimental replicates. (**D**) Spindle lengths and widths were measured for spindles of each stage, and either grouped by methodology (synched vs non-synced) or pooled (all). The number of spindles measured is shown (#), and average lengths and widths are given with SD. (**E**) A spindle (from experiments like those in A) lying perpendicular to the coverslip was imaged using confocal microscopy, and deconvolved. The image represents a single z plane at the midpoint of the spindle. (**F**) A cell (from experiments like those in A) with an apparent spindle irregularity reveals each bundle may be composed of multiple microtubules. A single z plane is shown. (**G**) Populations of synchronized cells were fixed and stained for tubulin (green) and DNA (magenta), and cells lying in the plane of the coverslip were imaged using a spinning disk confocal microscope. Sum intensity projections (center panels) were generated in Fiji, and line scans (right panels) were drawn from pole to pole (thick line), with a line thickness adjusted to encompass the entire spindle width (thinner box). The center of the spindle was set to 0 µm. (**H**) Line scans were drawn on spindles in prophase (left), metaphase (center), and anaphase/telophase (right). The pixel intensities along the spindle length were normalized to the average intensity of an area in the cell adjacent to the spindle, which was set to 1. Data are from 2 experimental replicates.

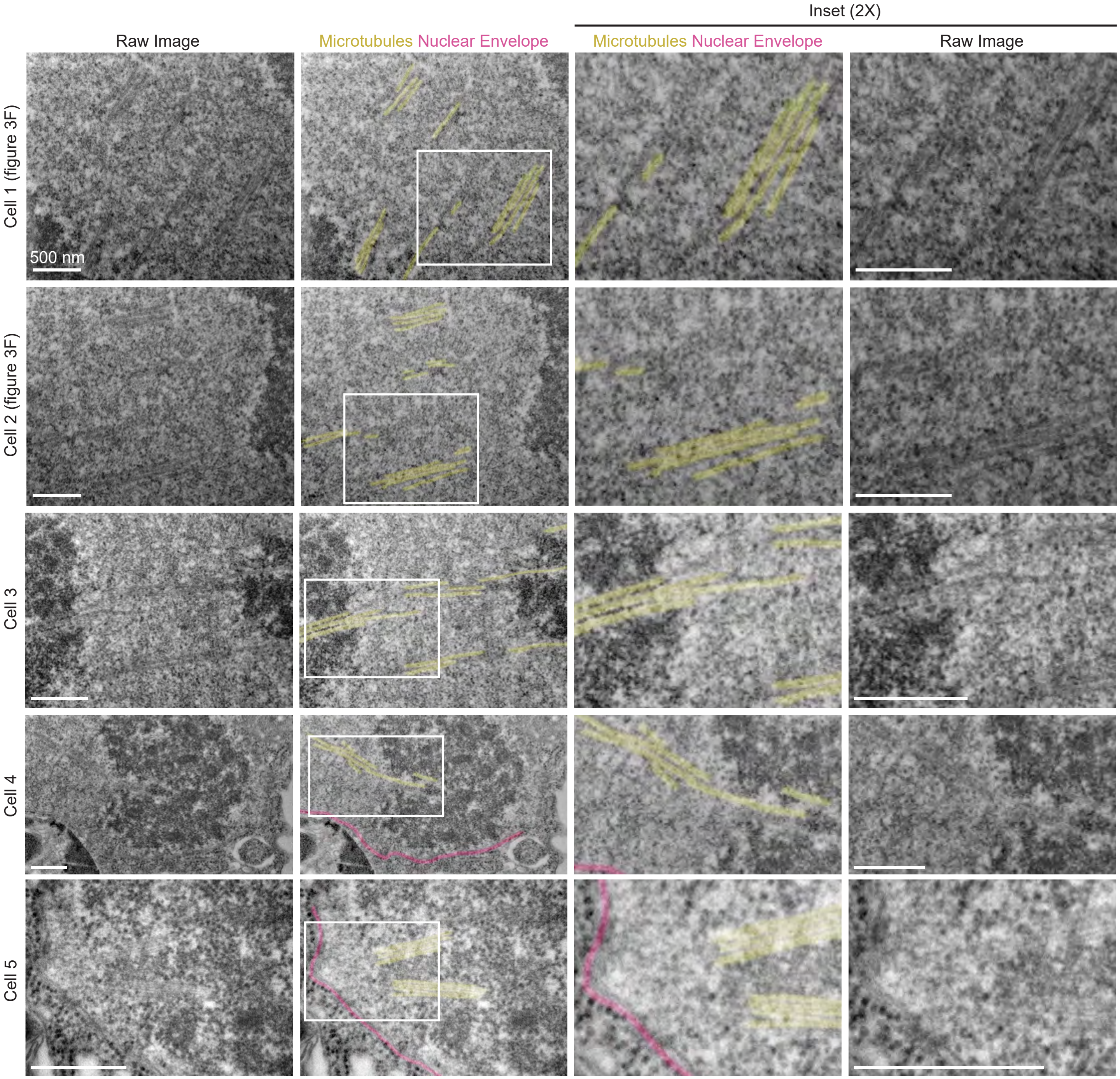

Figure S5. Additional electron micrographs of *Naegleria* spindles. Related to Figure 3. Transmission electron microscopy was used to examine microtubule bundles in dividing cells. Examples from 5 cells are shown; cells 1 and 2 correspond to **Figure 3F**. Microtubules are highlighted in yellow, and the nuclear envelope is highlighted in pink.

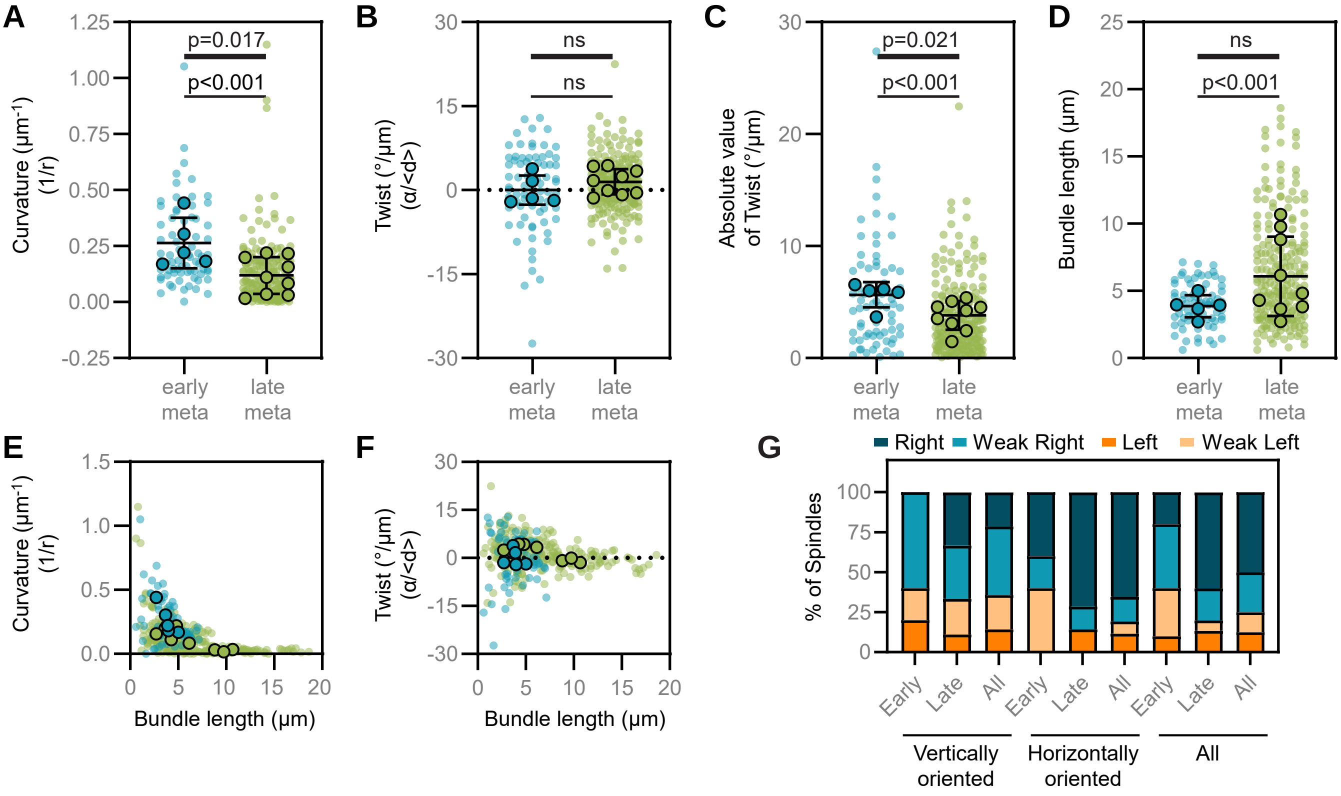

H

Stage	Curvature (1/μm)	Twist (°/μm)	Bundle length (μm)	Spindle length (μm)	Number of bundles	Number of spindles
**Early metaphase**	0.250 ± 0.021	0.059± 0.847	3.96 ± 0.18	7.19 ± 0.78	75	5
**Late metaphase**	0.111 ± 0.009	1.143 ± 0.313	6.43 ± 0.26	14.55 ± 2.56	226	9
**All metaphase**	0.146 ± 0.009	0.873 ± 0.316	5.81 ± 0.21	11.92 ± 1.90	301	14

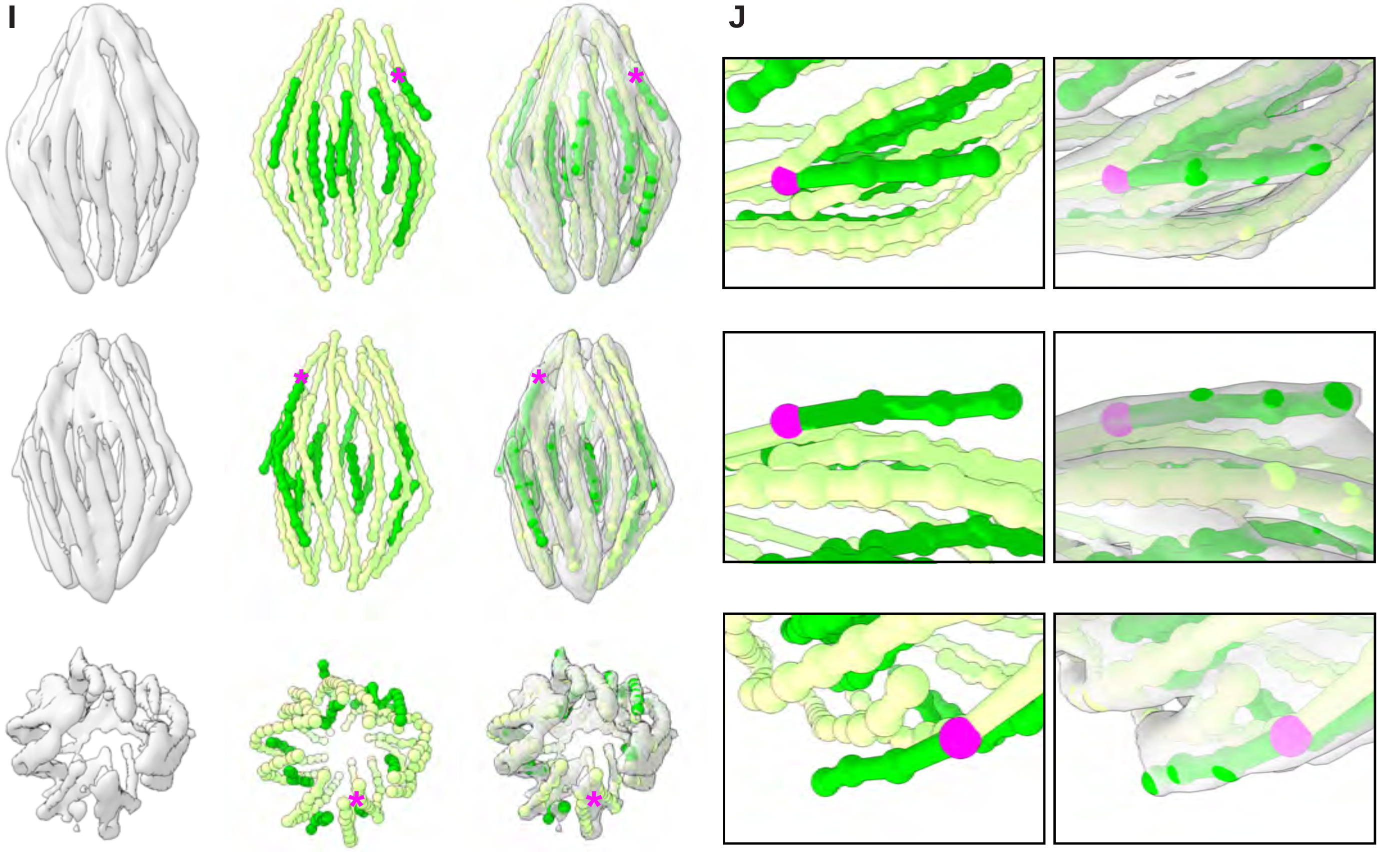

Figure S6. Curvature, twist, and arrangement of microtubule bundles. Related to Figure 4 and Figure 5. (**A**) Curvatures were calculated for individual bundles (smaller data points) and averaged for each spindle (larger data points). Lines indicate the mean and standard deviation calculated from spindle averages. Early metaphase bundles are significantly more curved when analyzed per bundle (indicated by thin line, Mann-Whitney test), or when spindle averages are compared (indicated by thick line, unpaired t test). (**B**) Bundle twists were calculated and compared, and are displayed as in A. The mean twist is different from 0 in late metaphase ($p= 0.0003$), but not in early metaphase ($p= 0.94$). To determine whether spindles are statistically different in early versus late metaphase, we compared the twist of both individual bundles and whole spindles and found no statistically supported difference (individual bundles: $p= 0.233$, unpaired t test, whole spindles: $p= 0.325$, unpaired t test). (**C**) The twist values in panel B were converted to absolute values. These values support more total twist in early metaphase than late for individual bundles (Mann-Whitney test) and whole spindles (unpaired t test). (**D**) Bundle lengths were measured and data are displayed for individual bundles and spindle averages as in A. Late metaphase spindles have longer bundles when all individual bundles are considered (Mann-Whitney test), but not when averaged by spindle ($p= 0.061$, unpaired t test). (**E**) The curvature of microtubule bundles is shown as a function of bundle length. Each small dot represents a single bundle within a spindle, while each larger dot represents the average for a spindle. Teal dots indicate bundles within early metaphase spindles, while green dots indicate late metaphase. (**F**) The twist of microtubule bundles is shown as a function of bundle length. Each small dot represents a single bundle within a spindle, while each larger dot represents the average for a spindle. Teal dots indicate bundles within early metaphase spindles, while green dots indicate late metaphase. For panels A-F, data were quantified from 4 experimental replicates, encompassing 14 cells and 301 bundles. (**G**) The percentage of spindles with right, weak right, left, or weak left handedness are shown. Spindles are grouped according to the stage of mitosis (early or late metaphase) and their orientation (vertical or horizontal), as indicated. The twist was determined visually by moving through end-on z-stacks from the bottom plane towards the top plane, where bundle rotation clockwise and counterclockwise implies a left-handed and right-handed twist, respectively. For horizontally oriented spindles, z-stacks were first rotated to obtain the end-on view. Data were taken from 4 experimental replicates. (**H**) The mean values (+/- SEM) for the bundle characteristics quantified above are shown. (**I**) A spindle from **Figure 4A** was rendered using ChimeraX, and primary and secondary bundles were traced using markers placed inside the 3D volume of each bundle. Longer bundles that persisted to the poles (primary bundles) were labeled with light green markers, while shorter bundles present at the midplane (secondary bundles) were labeled with dark green markers. Three angles of the same spindle are shown (top, middle, bottom) as volume renderings (left), markers (middle) and an overlay (right). (**J**) One point of close proximity between a secondary and primary bundle is highlighted as an example (the secondary bundle contains a magenta marker at this site, also see the magenta asterisk in panel A for context). Views from a variety of angles reveal that this secondary bundle also approaches other primary microtubule bundles.

Protein type	Kinesin Family	Kinesin Subfamily	JGI ID	NCBI Accession	Log2 fold change amoebae (+) vs flagellate (-)	Alternative gene models
kinesin	1		68781	XP_002676147.1	-3.637	34430
kinesin	1		72809	XP_002672080.1	0.256	
kinesin	1		79591	XP_002677830.1	-5.398	33335
kinesin	2		63939	XP_002680751.1	-7.189	31023
kinesin	2		88151	XP_002668333.1	-3.029	76756
kinesin	3		31717	XP_002680148.1	-5.887	
kinesin	3		31878	XP_002679701.1	2.728	
kinesin	3		65195	XP_002679451.1	0.075	3036
kinesin	3		69503	XP_002675395.1	-6.801	80350
kinesin	3		80962	XP_002673389.1	-9.768	
kinesin	3		88153	XP_002677049.1	-5.261	39261
kinesin	3		88158	XP_002676305.1	-0.528	68616
kinesin	5		64647	XP_002679930.1	1.729	31750
kinesin	6		69726	XP_002675105.1	-0.140	
kinesin	7		56509	XP_002683191.1	-8.585	
kinesin	7		69788	XP_002675060.1	0.942	
kinesin	7		74311	XP_002670651.1	-4.474	
kinesin	8		80478	XP_002674866.1	-2.405	
kinesin	8		81903	XP_002670277.1	-0.465	
kinesin	9		47171	XP_002679918.1	-2.348	31675
kinesin	9		64648	XP_002680042.1	-6.730	
kinesin	13		64960	XP_002679830.1	-1.082	31834
kinesin	13		79173	XP_002678989.1	-7.088	32337
kinesin	13		88159	XP_002681280.1	0.457	63167
kinesin	14	A	65526	XP_002679253.1	2.259	32240
kinesin	14	A	73429	XP_002671391.1	-7.743	
kinesin	14	B	66196	XP_002678643.1	-1.409	32757
kinesin	14	B	75257	XP_002669853.1	-2.010	60999
kinesin	14	B	78071	XP_002682529.1	-7.729	30037
kinesin	15		79295	XP_002678628.1	1.444	2164
kinesin	15		79561	XP_002677693.1	1.929	
kinesin	16		71374	XP_002673531.1	-3.702	
kinesin	Unknown		61244	XP_002683093.1	0.001	5496
kinesin	Unknown		61291	XP_002683123.1	0.041	61291
kinesin	Unknown		63602	XP_002681066.1	-0.690	5502
kinesin	Unknown		71346	XP_002673441.1	-4.472	
kinesin	Unknown		71914	XP_002672926.1	2.573	
kinesin	Unknown		76829	XP_002668262.1	0.193	76829
kinesin	Unknown		78506	XP_002681223.1	2.497	
kinesin	Unknown		79563	XP_002677796.1	1.659	79563
kinesin	Unknown		82323	XP_002668732.1	-0.825	
kinesin	Unknown		88162	XP_002675693.1	3.094	88162
cytoplasmic dynein	NA	NA	46538	XP_002681057.1	-3.732	
Unclassified dynein motor chain	NA	NA	61303	XP_002683130.1	-6.268	

Table S1. Relative mRNA expression of microtubule motor proteins. Related to Figure 5. A table showing the relative mRNA expression of kinesin and cytoplasmic dynein heavy chain genes in flagellates (which do not divide) relative to amoebae (a fraction of which are dividing). Each kinesin was previously assigned to a family by phylogenetic analysis; genes marked “Unknown” were not clearly associated with a known kinesin family as of the time of original publication.[S1] The JGI and NCBI accession numbers for each gene are provided, including alternative JGI IDs corresponding to a different gene model for the same genetic locus. Gene expression differences are indicated by the log2-transformed fold-change between expression levels in amoebae (a sample taken just prior to amoebo-flagellate differentiation) relative to expression levels in flagellates (a sample taken 80 minutes post differentiation). Expression data is the average of three biological replicates. Positive values indicate higher expression in amoebae; negative values indicate higher expression in flagellates. Data are from Fritz-Laylin and Cande, 2010.[S2]

Figure Number	Figure Panel	Number of experiments	Total number of cells or technical replicates	Synched or non-synched?
1	D	3	6	N/A
3	B	3	20	non-synched
4	B	3	8	synched and non-synched
4	C	4	21	synched and non-synched
4	D	2	15 representatives shown	non-synched
4	E	4	52	synched and non-synched
5	C	4	14 cells (301 bundles)	synched and non-synched
5	D	4	14 cells (301 bundles)	synched and non-synched
5	E	4	40 cells	synched and non-synched
S1	A	5	5	synched
S1	B	2	6	synched
S4	B	2	35	synched
S4	C	4	29	synched and non-synched
S4	H	2	12 representatives shown	synched
S6	A	4	14 cells (301 bundles)	synched and non-synched
S6	B	4	14 cells (301 bundles)	synched and non-synched
S6	C	4	14 cells (301 bundles)	synched and non-synched
S6	D	4	14 cells (301 bundles)	synched and non-synched
S6	E	4	14 cells (301 bundles)	synched and non-synched
S6	F	4	14 cells (301 bundles)	synched and non-synched
S6	G	4	40 cells	synched and non-synched

Table S2. The number of experiments, cells, or microtubule bundles used for each analysis. Related to Figures 1-5. The number of experimental replicates (different days the entire experiment was completed) and technical replicates (the number of cells or microtubule bundles) are listed for each figure panel of the manuscript.

Supplemental References

S1 Fritz-Laylin, L.K., Prochnik, S.E., Ginger, M.L., Dacks, J.B., Carpenter, M.L., Field, M.C., Kuo, A., Paredez, A., Chapman, J., Pham, J., et al. (2010). The genome of Naegleria gruberi illuminates early eukaryotic versatility. Cell 140, 631–642.

S2 Fritz-Laylin, L.K., and Cande, W.Z. (2010). Ancestral centriole and flagella proteins identified by analysis of Naegleria differentiation. J. Cell Sci. 123, 4024–4031.
