# Supplementary material for: Naegleria’s mitotic spindles are built from unique tubulins and highlight core spindle features
Source: Curr Biol. Author manuscript; Available in PMC 2022 Apr 25. (PMC9036621; doi:10.1016/j.cub.2022.01.034)
Supplement: Data S2 [file NIHMS1779381-supplement-Data_S2.pdf]

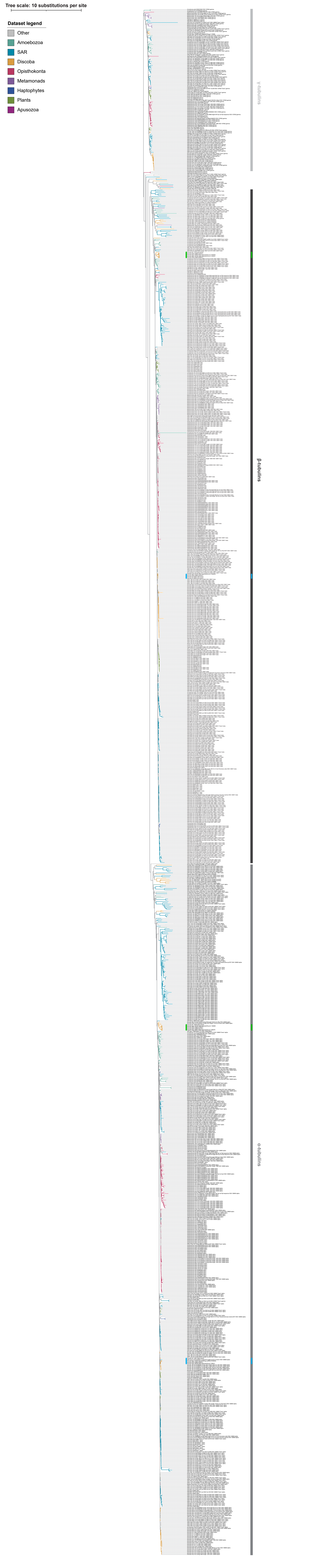
Tree scale: 10 substitutions per site
Dataset legend
Other
Amoebozoa
SAR
Discoba
Opisthokonta
Metamonads
Haptophytes
Plants
Apusozoa
γ-tubulins
β-tubulins
α-tubulins
